# Expression of the vault RNA protects cells from undergoing apoptosis

**DOI:** 10.1038/ncomms8030

**Published:** 2015-05-08

**Authors:** Melanie Amort, Birgit Nachbauer, Selma Tuzlak, Arnd Kieser, Aloys Schepers, Andreas Villunger, Norbert Polacek

**Affiliations:** 1 Division of Genomics and RNomics, Medical University Innsbruck, Innsbruck A-6020, Austria; 2 Department of Chemistry and Biochemistry, University of Bern, Bern CH-3012, Switzerland; 3 Graduate School for Cellular and Biomedical Sciences, University of Bern Bern 3012, Switzerland; 4 Division of Developmental Immunology, Medical University Innsbruck, Innsbruck A-6020, Austria; 5 Research Unit Gene Vectors, Helmholtz Zentrum München, München D-81377, Germany; 6 German Center for Infection Research (DZIF), Partner site Munich, München D-81377, Germany

## Abstract

Non-protein-coding RNAs are a functionally versatile class of transcripts exerting their biological roles on the RNA level. Recently, we demonstrated that the vault complex-associated RNAs (vtRNAs) are significantly upregulated in Epstein–Barr virus (EBV)-infected human B cells. Very little is known about the function(s) of the vtRNAs or the vault complex. Here, we individually express latent EBV-encoded proteins in B cells and identify the latent membrane protein 1 (LMP1) as trigger for vtRNA upregulation. Ectopic expression of vtRNA1-1, but not of the other vtRNA paralogues, results in an improved viral establishment and reduced apoptosis, a function located in the central domain of vtRNA1-1. Knockdown of the major vault protein has no effect on these phenotypes revealing that vtRNA1-1 and not the vault complex contributes to general cell death resistance. This study describes a NF-κB-mediated role of the non-coding vtRNA1-1 in inhibiting both the extrinsic and intrinsic apoptotic pathways.

## Abstract (web summary)

Cellular functions of the vault complex, a large ribonucleoprotein assembly remain elusive. Here, the authors show that Epstein–Barr virus infection enhances the expression of the vault complex-associated RNAs, which leads to improved survival of infected cells due to the inhibition of cell apoptosis.

## Graphical abstract

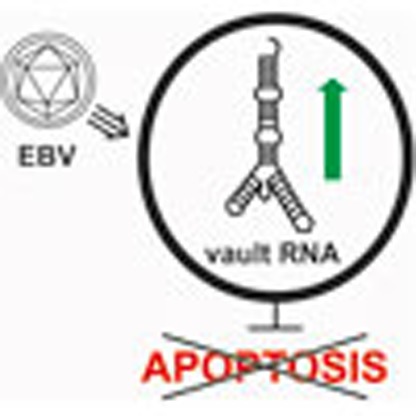

Non-protein-coding RNAs (ncRNAs) function on the level of RNA and are not translated into proteins. ncRNAs are prevalent in regulating many important cellular processes in all domains of life^1^. Initially, ncRNAs were suggested to only play functional roles in protein synthesis as integral components (ribosomal RNA) or reaction substrates (transfer RNA) of the ribosome but over the years multiple additional functions were identified. They are involved in regulating a diversity of fundamental processes including transcription, translation, RNA processing, mRNA turnover, DNA replication, genome stability, chromatin remodelling and even contribute to the stability and location of proteins^1^,^2^,^3^,^4^,^5^. Due to the power of deep-sequencing methods, more comprehensive insights into cellular transcriptomes became possible and emphasized that multicellular eukaryal organisms possess significantly more ncRNA genes compared with more primitive single-cell eukaryotes (for example, yeast) or prokaryotes. These results led to the hypothesis, that ncRNAs can establish intricate regulatory networks and may be key to understanding the increased complexity of mammals compared with ‘lower organisms’, despite the only modestly higher number of protein-coding genes^6^.

However, many of the cellular ncRNA transcripts lack experimental confirmation of their biological role. Even though the class of vault RNAs (vtRNAs) have been initially identified almost 30 years ago^7^, its function is not yet completely clear. The vtRNAs have been identified as integral components of the vault complex, a hollow barrel-shaped ribonucleoprotein (RNP) complex with a size of 13 MDa found in most eukaryotes^8^. This gigantic complex is by far the largest cellular RNP identified to date and several functions have been suggested for the vault complex. These include roles in nucleocytoplasmic transport^9^, intracellular detoxification processes and hence in multidrug resistance of cancer cells^10^,^11^, signalling^12^,^13^, apoptosis resistance^14^, innate immune response^15^, DNA damage repair^16^ and recently also in nuclear pore complex formation^17^. In addition to the vtRNAs, the vault complex consists of multiple copies of three proteins: the major vault protein (MVP), the vault poly(ADP-ribose)-polymerase (vPARP) and the telomerase-associated protein 1 (TEP1). MVP is the major structural protein of the vault complex, contributes with ∼70% to the particles mass and self-assembles to form vault-like particles *in vivo*^18^. In humans, four vtRNA paralogues, vtRNA1-1, vtRNA1-2, vtRNA1-3 and vtRNA2-1, have been detected^8^,^19^. They are 88–100 nucleotides in length, are clustered on chromosome 5, and show little sequence conservation between species except their A and B boxes, which are internal polymerase III elements. The stoichiometric model of the vault particle proposes that it contains at least six copies of vtRNA which, however, comprise <5% of the total vault complex mass. Intriguingly, only 5% of the vtRNAs are directly associated to the vault particle, whereas the remaining 95% are evenly distributed throughout the cytoplasm^10^,^19^,^20^. In addition, minute nuclear vtRNA localization has been reported as well^21^.

Recently, we observed that vtRNAs were upregulated in human B cells upon Epstein–Barr virus (EBV) infection^19^,^22^. EBV belongs to the γ-herpesvirus family and is involved in the development of aggressive lymphomas and carcinomas, such as Burkitt lymphoma (BL), Hodgkins lymphoma and nasopharyngeal carcinoma^23^. Three main types of EBV latency stages are known (latency I–III), which are characterized by the expression of a distinct set of viral genes depending on the infected cell type^24^. *In vitro* infection of the EBV-negative lymphoid cell line BL2 establishes a latency III pattern, characterized by the expression of nine EBV proteins (EBNA1, EBNA2, EBNA3a, 3b, 3c, EBNA-LP, LMP1, LMP2a and LMP2b), two ncRNAs (EBER1 and EBER2) and several miRNAs (BART and BHRFs)^25^,^26^. Hence, there seems to be a causal link between the presence of EBV and the upregulation of cellular vtRNAs^19^.

In this work, we individually overexpress most latent EBV-encoded proteins and identify LMP1 as trigger for NF-κB-dependent vtRNA1-1 expression. Ectopic expression of vtRNA1-1 in a B-cell line that usually lacks this ncRNA renders the cells amenable to efficient EBV infection by protecting them from undergoing apoptosis. Knockdown experiments of the MVP reveals that the anti-apoptotic effect is a function of vtRNA1-1 not associated with the genuine vault complex. While the MVP has been previously suggested to inhibit apoptosis in senescent cells^14^, this study is the first report demonstrating general apoptotic resistance upon vtRNA1-1 expression in malignant B cells.

## Results

### LMP1 stimulates vtRNA1-1 expression in BL2 cells

**Figure 1:**
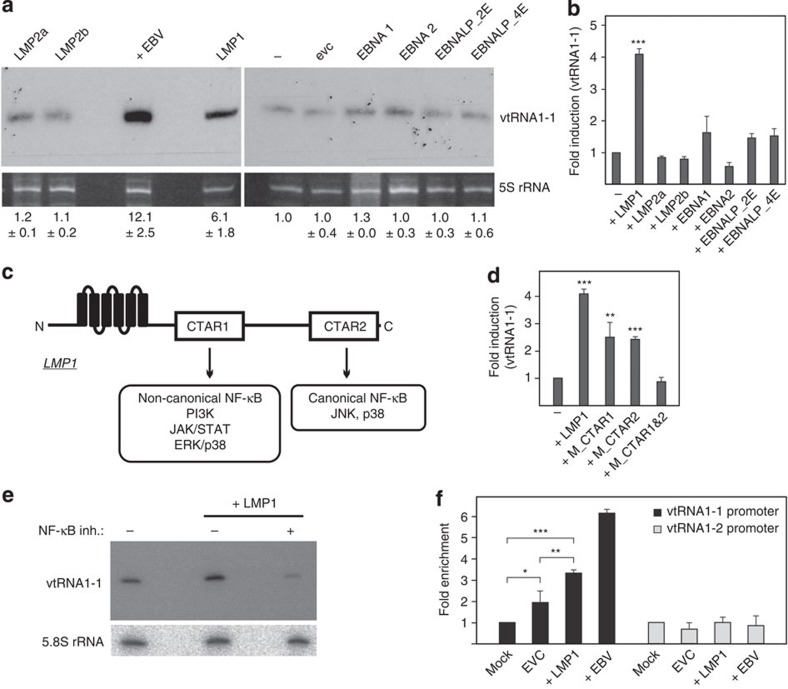
vtRNA levels in response to expression of EBV-encoded latency III proteins. (**a**) Northern blot analysis was used to assess the effects of overexpressing EBV-encoded proteins on the vtRNA1-1 levels in different BL2 cell lines. The 5S rRNA serves as internal loading control. (−) indicate untreated BL2 cells, whereas EVC indicates the empty vector control. EBV strain B95.8 was used as positive infection control (+ EBV). The mean and standard deviation of three different experiments are shown beneath the blot, whereas the vtRNA1-1 expression levels in untreated BL2 cells (−) was taken as 1.0. See also Supplementary Fig. 2. **(b)** Real-time qPCR was used to quantify vtRNA1-1 levels in the untreated BL2 control (−) or in BL2 cells overexpressing latency stage III proteins. Real-time qPCR data shown are the mean values from three individual assays; TBP served as housekeeping gene. ‘Fold induction’ was calculated using the comparative Δct method where BL2 served as calibrator. **(c)** Schematic representation of LMP1 domain organization. The different signalling pathways controlled by the signalling modules CTAR1 and CTAR2 are indicated. (**d)** Real-time qPCR to investigate the effect of LMP1 mutants (M_CTAR 1 and M_CTAR 2 carry detrimental amino-acid substitutions or a deletion in CTAR domains 1 or 2, respectively) on the vtRNA1-1 level in BL2 cells. Data shown are the mean values and standard deviations of three independent assays. (**e)** In the presence of an NF-κB inhibitor (inh.), northern blot analysis showed no LMP1-dependent upregulation of vtRNA1-1. 5.8 S rRNA serves as internal loading control. **(f**) ChIP efficiencies using an NF-κB p65 antibody were monitored using qPCR on vtRNA1-1 and vtRNA1-2 promoter regions from BL2 cells carrying the empty vector (evc), expressing LMP1 (+LMP1), or from EBV-infected BL2 cells. Data shown are mean values and standard deviations from three individual assays. Significant differences relative to the mock control (no antibody) were determined using the two-tailed unpaired Student’s *t*-test (***P<0.001, **P<0.01, *P<0.01). In **b** and **d**, significant differences in fold induction relative to the untreated BL2 cells (−) were determined using the two-tailed unpaired Student’s *t*-test (***P<0.001, **P<0.01).

Previously, we have shown that EBV infection of Burkitt lymphoma BL2 or BL41 cells leads to a significant upregulation of human vtRNA1-1 and vtRNA2-1, or vtRNA2-1, respectively^19^,^22^. To determine which EBV-encoded gene product triggers this host cell response, we individually overexpressed latency phase III proteins in BL2 cells^25^,^26^. By using stable lentiviral transduction, we successfully expressed the EBV proteins EBNA1, EBNA2, EBNA-LP, LMP1, LMP2a and LMP2b in these cells (Supplementary Fig. 1). The EBNA3-A, -B, -C family was not included in this study since its function is predominately transcriptionally repressive^27^,^28^. Subsequent to EBV-protein expression the vtRNA levels were analysed by northern blot analysis (Fig. 1a, Supplementary Fig. 2) and by quantitative real-time PCR (Fig. 1b). These analyses revealed that only LMP1 significantly contributed to vtRNA1-1 upregulation (up to 6-fold), while the expression of the other EBV-encoded proteins LMP2a, LMP2b, EBNA1, EBNA2 and EBNA-LP had little or no effect (Fig. 1a,b). This effect is specific for vtRNA1-1 since no upregulation of the other vtRNA paralogues was observed (Supplementary Fig. 3a). This LMP1-dependent expression of vtRNA1-1 was also evident in several other B cell lymphoma lines (Supplementary Figs 3b and 4c,d). LMP1, a six-transmembrane domain protein, is the principal EBV oncogene and is essential for efficient B-cell transformation^29^. It influences host gene expression through the activation of different signalling pathways. The cytoplasmic C-terminal activator regions (CTAR) 1 and 2 associate with cellular signaling molecules and stimulate the following pathways: CTAR1 is involved in triggering the non-canonical NF-κB pathway, the PI3K and the JAK/STAT pathways, and the MAPK pathways ERK, p38 and in some cell lines, JNK. CTAR2 is known for activating the canonical NF-κB pathway and the MAPK pathways JNK and p38 (Fig. 1c). To gain deeper insight into the LMP1-mediated vtRNA upregulation, LMP1 mutants with individually inactivated CTAR domains (M_CTAR1 representing a P204 × Q × T → A × A × A mutation, or M_CTAR2 harbouring a deletion of the C-terminal residues 371–386), or a variant carrying both CTAR mutations simultaneously (M_CTAR1&2) were expressed in BL2 cells (Supplementary Fig. 1). Quantitative real-time PCR demonstrated that both single CTAR mutants retained activities in vtRNA1-1 upregulation, while the double mutant (M_CTRAR1&2) has lost its potential to stimulate expression of vtRNA1-1 (Fig. 1d). This activity profile of the LMP1 mutants is compatible with the notion that LMP1 activates vtRNA1-1 expression through both the canonical and the non-canonical NF-κB pathways. In support of this interpretation, repeating the experiment in the presence of the IKK-VII inhibitor at concentrations efficiently blocking IKK-α, central to both NF-κB pathways, completely abolished vtRNA1-1 upregulation in LMP1 expressing BL2 cells (Fig. 1e). Consistent with LMP1-driven NF-κB activation, the upregulation of three NF-κB target genes, that is, Bcl-xL, IL6 and c-FLIP was observed (Supplementary Fig. 3c). To determine a direct interaction of the dominant NF-κB member p65/RelA with the vtRNA1-1 promoter, we performed NF-κB chromatin immunoprecipitation (ChIP) experiments (Fig. 1f). According to CHIP-seq data of the ENCODE project, a NF-κB binding site was predicted within the promotor region of vtRNA1-1 (refs 30, 31). Indeed, our ChIP experiments revealed a significant enrichment of the vault RNA1-1 promoter in LMP1-overexpressing or EBV infected BL2 cells. We observed a minor enrichment in the empty vector control, indicative for basal promoter occupancy by p65/RelA under steady state conditions (Supplementary Fig. 3d), suggesting that the weak endogenous vtRNA1-1 levels in the parental BL2 cell line were also NF-κB-dependent (Fig. 1a). In accordance with the ENCODE project, we also did not observe any enrichment of the vtRNA1-2 promoter region, highlighting the specificity of the ChIP assay (Fig. 1f). In summary, these experiments provide evidence that NF-κB is involved in regulating the vtRNA1-1 but not the vtRNA1-2 promoter.

### Ectopic expression of vtRNA1-1 stimulates EBV establishment

**Figure 2:**
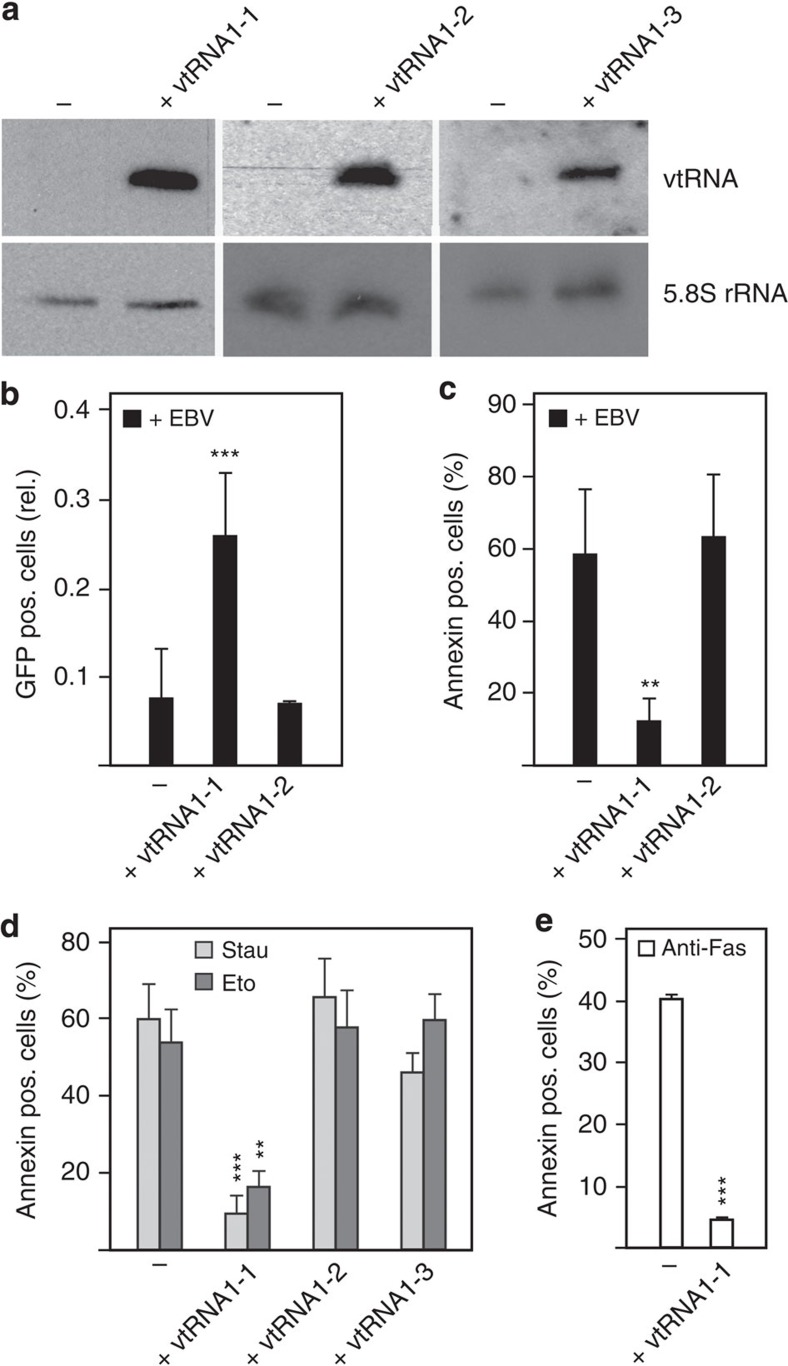
Effect of vtRNA levels on EBV infection and apoptosis. (**a**) Northern blot analysis reveals the amount of ectopically expressed vtRNA1-1, 1-2 and 1-3 in BL41 cells. The 5.8S rRNA serves as internal loading control. See also Supplementary Fig. 5. (**b**) Fluorescence-activated cell sorting (FACS) analysis to investigate the effect of infecting the BL41 cell line with the recombinant EBV virus 2089 (ref. 32) in the absence (−) or presence of ectopically expressed vtRNA1-1 or vtRNA1-2. The data shown represent the mean and standard deviation of 11 independent experiments. The GFP-positive (pos.) Raji cells after EBV infection (infection efficiency typically 30%) served as positive infection control and were taken as 1.0. **(c)** The same cells as in **b** were labelled with annexin V PE and apoptotic cells were recorded on FL-2. **(d)** BL41 cells without (−) or with vtRNA1-1, 1-2 or 1-3 expression were treated with staurosporine (Stau) or etoposide (Eto) and the amount of apoptotic cells was determined by FACS analysis after an annexin stain. The data originates from five independent experiments. The number of apoptotic cells in the untreated control was subtracted in each individual experiment from the staurosporine- or etoposide-treated ones. The numbers of apoptotic cells in the untreated controls were <12%. **(e)** BL41 cells without (−) or with vtRNA1-1 expression were treated with anti-Fas antibody and the amount of apoptotic cells determined. The mean and standard deviations of two independent experiments are shown. The numbers of apoptotic cells in the untreated controls were <10% and were subtracted from each experiment. In **b**, **c**, **d** and **e**, significant differences relative (rel.) to the untreated control cells (−) were determined using the two-tailed unpaired Student’s *t*-test (***P<0.001, **P<0.01).

Our data suggest that LMP1 is the EBV-protein upregulating vtRNA1-1 in BL2 cells. We next addressed the question whether this host ncRNA has an impact on the EBV infection process. To study the putative causal link between EBV infection and vtRNA1-1 upregulation, we ectopically expressed vtRNAs in human B cells and tested the consequences on EBV infection. For these experiments, the BL41 cell line was used since EBV infection normally does not trigger detectable vtRNA1-1 levels in this cell line^19^. Thus the effects of ectopically expressed vtRNA1-1 on EBV infection can be studied without any detectable endogenous vtRNA background expression. Stably transfected BL41 cell lines were established expressing vtRNA1-1 or the paralogues vtRNA1-2 and vtRNA1-3 (Fig. 2a, Supplementary Fig. 5). Subsequently, these cell lines were infected with the recombinant GFP expressing EBV variant 2089 (ref. 32) and the number of GFP-positive cells was determined by fluorescence-activated cell sorting analysis 72 h post infection. The vtRNA1-1 overexpressing cell line showed a markedly enhanced viral establishment rate (Fig. 2b). In fact, the empty vector control cell line, which does not express detectable levels of vtRNA1-1, was almost resistant to EBV infection (Fig. 2b). Cells expressing the paralogue vtRNA1-2 behaved like the empty vector control cells and were poorly infected by the virus (Fig. 2b), thus hinting at a vtRNA1-1 specific effect. The enhanced EBV establishment can be partly explained by the fact that the vtRNA1-1 overexpressing cell line exhibited an accelerated growth rate compared with the parental BL41 cell line (Supplementary Fig. 4a). However, this phenotype was not linked to an altered cell cycle profile in the vtRNA1-1 overexpressing cell line (Supplementary Fig. 4b). Furthermore, the enhanced virus establishment likely originates from the decreased cell death rate of the BL41 cells expressing vtRNA1-1 in the presence of EBV (Fig. 2c).

### vtRNA1-1 expression confers apoptosis resistance

**Figure 3:**
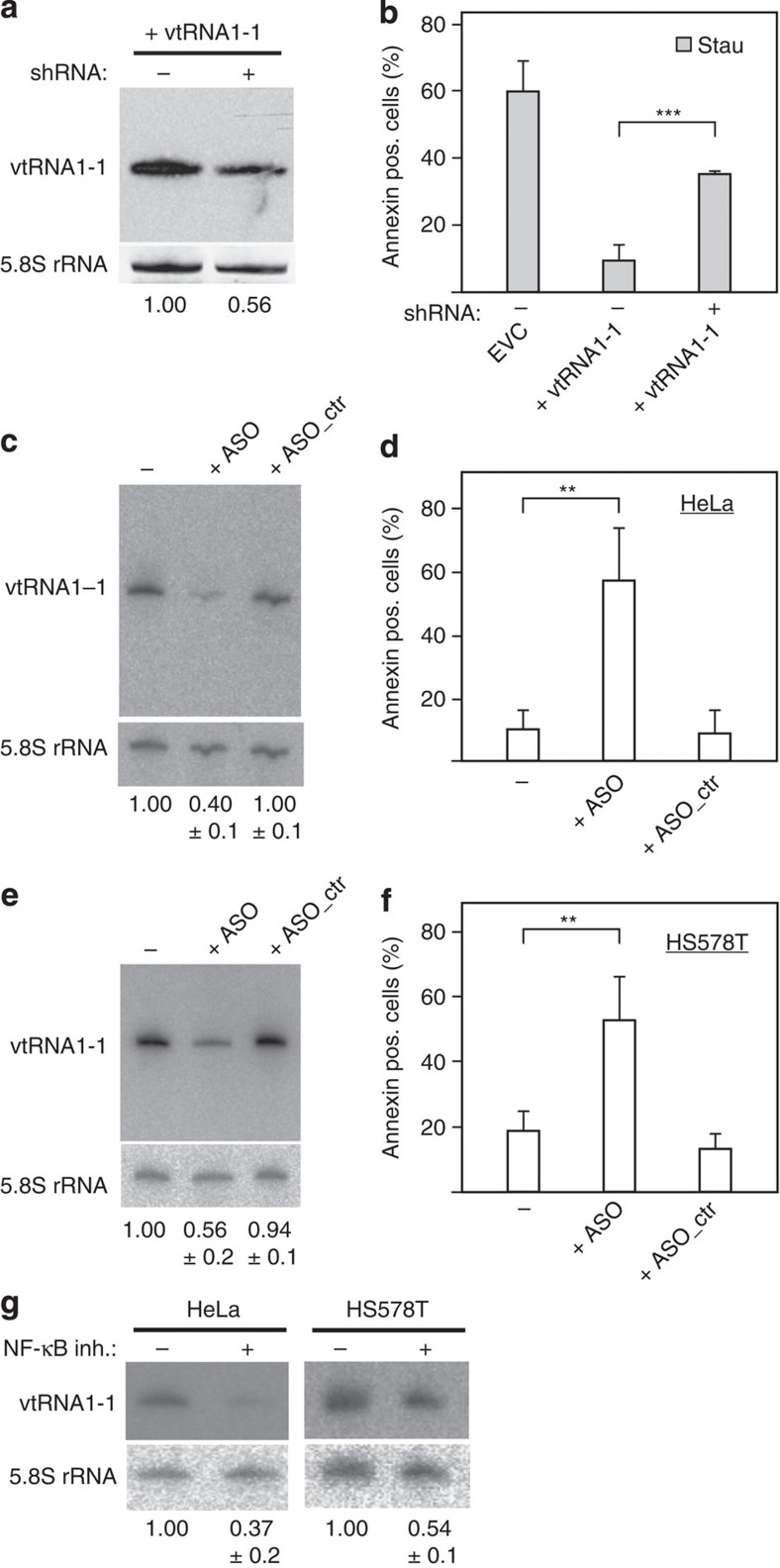
Dose dependence of vtRNA1-1 levels on the impact of apoptosis resistance. (**a**) Northern blot analyses demonstrate an shRNA-mediated knockdown of vtRNA1-1 levels in BL41 cells of ∼50%. See also Supplementary Fig. 6. **(b)** Annexin V staining revealed a reduced apoptosis resistance in the vtRNA1-1 knockdown BL41 cell line after staurosporine (Stau) treatment. The knockdown has been performed in two biological replicates whereas the mean and standard deviations of four technical replicates are shown. **(c)** Northern blot analysis showed an ∼60% reduction of endogenous vtRNA1-1 levels in HeLa cells upon transfection of an anti-sense oligonucleotide analogue (ASO). Administration of a control oligonucleotide (ASO_ctr) strand had no effect. (−) depict HeLa cells that were treated as the transfected samples, but in the absence of any oligonucleotides. Quantifications of band intensities are shown below the blot and represent the mean and standard deviation of three independent transfection experiments. The 5.8S rRNA served as internal loading control. **(d**) Reduced endogenous vtRNA1-1 levels result in an increased susceptibility to cell death in HeLa cells. The mean and standard deviations of three independent transfection experiments are shown. (**e**,**f**) Analogous to **c** and **d**, dose-dependent effects on apoptosis, resistance as a function of vtRNA1-1 concentration were monitored in the human breast cancer cell line HS578T. **(g)** In the presence of the NF-κB inhibitor (inh.) IKK VII, northern blot analysis showed a decreased vtRNA1-1 expression level in HeLa and HS578T cells. The 5.8S rRNA serves as internal loading control. The endogenous levels of vtRNA1-1 in HeLa and HS578T cells was taken as 1.0 and compared with cells treated with a NF-κB inhibitor. In **b**, **d** and **f**, significant differences were determined using the two-tailed unpaired Student’s *t*-test (***P<0.001, **P<0.01). pos., positive.

To unravel the reason for the observed increased EBV establishment in vtRNA1-1 overexpressing cell lines, the fraction of apoptotic cells in the culture was determined by Annexin V staining. Indeed, expression of vtRNA1-1, but not vtRNA1-2, or vtRNA1-3, protected BL41 cells from undergoing apoptosis after EBV infection (Fig. 2c). To examine the role of vtRNA1-1 in general apoptosis resistance, we challenged the vtRNA overexpressing cell lines with two well-known inducers of intrinsic programmed cell death, namely the pan-kinase inhibitor staurosporine or the topoisomerase-II inhibitor etoposide. Cells were treated for 24 h with either drug and subsequently subjected to the Annexin binding assay followed by flow cytometric analysis. BL41 cells overexpressing vtRNA1-1 exhibited a significantly reduced apoptosis rate compared with the control cell line (Fig. 2d). Again, expression of the paralogues vtRNA1-2 and vtRNA1-3 did not protect BL41 cells from undergoing cell death. In addition, only vtRNA1-1 protected BL41 cells from the Fas ligand-induced extrinsic apoptosis (Fig. 2e). The vtRNA-mediated apoptosis resistance was dosage dependent, since short hairpin RNA (shRNA)-mediated knockdown of vtRNA1-1 expression in BL41 cells reversed the cell death resistance phenotype. Reducing the vtRNA1-1 levels by about half resulted in a markedly increased apoptosis (Fig. 3a,b, Supplementary Fig. 6). Dose dependency of vtRNA1-1-triggered apoptosis resistance was also evident in BL2 cells (Supplementary Fig. 7). A similar effect was observed in HeLa cells and in the breast cancer cell line HS578T in which the endogenous vtRNA1-1 levels were reduced by ∼50% by an anti-sense oligonucleotide (ASO) approach^33^. Knockdown of vtRNA1-1 levels in these two cancer cell lines resulted in a clear increase of spontaneous cell death in culture (Fig. 3c–f). In both of these cancer cell lines, vtRNA1-1 expression appears to be also driven by NF-κB since the levels significantly decreased in the presence of the IKK inhibitor VII (Fig. 3g). Taken together, these findings indicate that vtRNA1-1 has an important impact on apoptosis resistance in different cancer cell types.

### vtRNA1-1 central domain is responsible for apoptosis resistance

**Figure 4:**
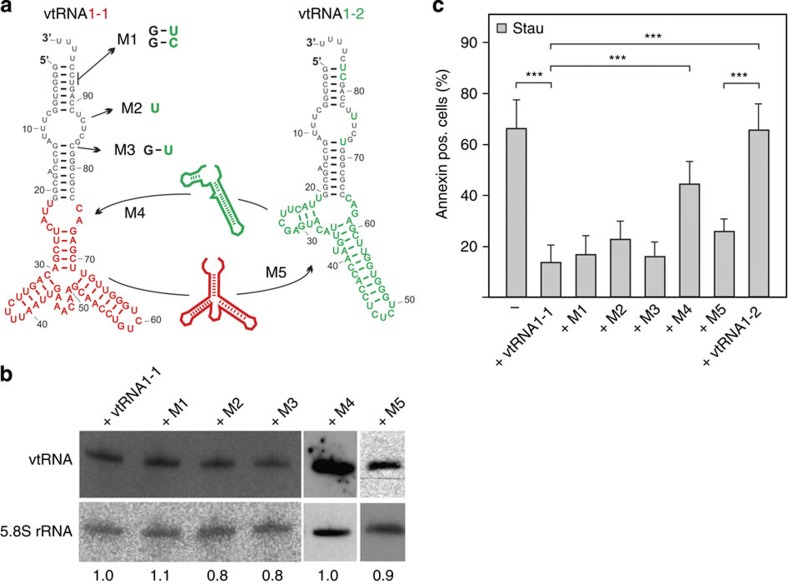
Mutational analyses highlight the importance of the central domain of vtRNA1-1 for apoptosis resistance. (**a**) Secondary structure models of vtRNA1-1 and vtRNA1-2, whereas the nucleotides that differ in vtRNA1-2 from vtRNA1-1 are depicted in green. M1-M3 depict vtRNA1-1 mutant constructs that carry vtRNA1-2 nucleobase identities at corresponding vtRNA1-1 positions. M4 represents a vtRNA1-1 mutant construct that carries the entire vtRNA1-2 central domain (green) instead of the corresponding vtRNA1-1 central domain (red). M5 depicts a vtRNA1-2 mutant that carries the entire central domain of vtRNA1-1. **(b)** The expression levels of M1 to M5 in BL41 were analysed by northern blot analyses with an oligonucleotide able to detect all vtRNA variants. Levels of ectopically expressed vtRNA1-1 in BL41 cells was set to 1.0 and compared with cells expressing M1–M5 mutant vtRNAs. The 5.8S rRNA served as internal loading control. See also Supplementary Fig. 8. (**c**) After a staurosporine (Stau) treatment, BL41 cells expressing the mutant vtRNAs were annexin V stained and apoptotic cells were recorded on FL2 on a fluorescence-activated cell sorting (FACS) device. Untreated BL41 cells without detectable vtRNA1-1 expression (−) or BL41 cells expressing the non-protective vtRNA1-2 served as apoptosis controls. The mean and the standard deviation of 10 independent experiments are shown. The numbers of apoptotic cells in the untreated controls were <10% and were always subtracted from the staurosporine-treated cells. Significant differences were determined using the two-tailed unpaired Student’s *t*-test (*** *P*<0.001). pos., positive.

The data described above showed that expression of vtRNA1-1 but not of the vtRNA1-2 or vtRNA1-3 paralogues confer resistance to apoptosis in different cell lines including BL41. The vtRNA paralogues are highly similar and differ only slightly in primary and secondary structure, especially in the terminal stem-loop region (Fig. 4a). The main differences between vtRNAs paralogues reside in the central domain^8^. To determine the specific sequence motif(s) of vtRNA1-1 contributing to the anti-apoptotic effect, vtRNA mutants were generated and expressed in BL41 cells (Fig. 4a,b, Supplementary Fig. 8). Therefore those nucleotides in vtRNA1-2 that differ from vtRNA1-1 in the terminal stem-loop domain (M1–M3) were introduced into the latter. Furthermore, the central domain of vtRNA1-2 was swapped into the corresponding region of vtRNA1-1 (M4). Transduced cells were treated with staurosporine for 24 h and the fraction of dead cells determined by Annexin V staining. It turned out that none of the vtRNA1-1 variants carrying base changes in the terminal stem-loop (M1–M3) has lost its principal ability to protect cells from undergoing apoptosis (Fig. 4c). However, swapping the central domain of vtRNA1-2 into the vtRNA1-1 molecule (M4) made BL41 cells again susceptible to the staurosporine treatment and reduced the ability of this transcript to protect cells from apoptosis (Fig. 4c). These data indicate the central domain of vtRNA1-1 (from residues 22–75) as the major determinant for the anti-apoptotic effect. To substantiate this interpretation, we constructed a ‘reverse-swap’ mutant (M5), in which the central domain of vtRNA1-1 was transplanted into the non-protective vtRNA1-2 paralogue. Indeed, expression of this chimeric vtRNA M5 protected cells from undergoing apoptosis (Fig. 4c). Thus the anti-apoptotic characteristics of vtRNA1-1 can be transplanted into the vtRNA1-2 backbone by swapping the central domain confirming that this domain of vtRNA1-1 is essential and sufficient to render BL41 cells staurosporine resistant.

### Apoptosis resistance is a function of the vtRNA

**Figure 5:**
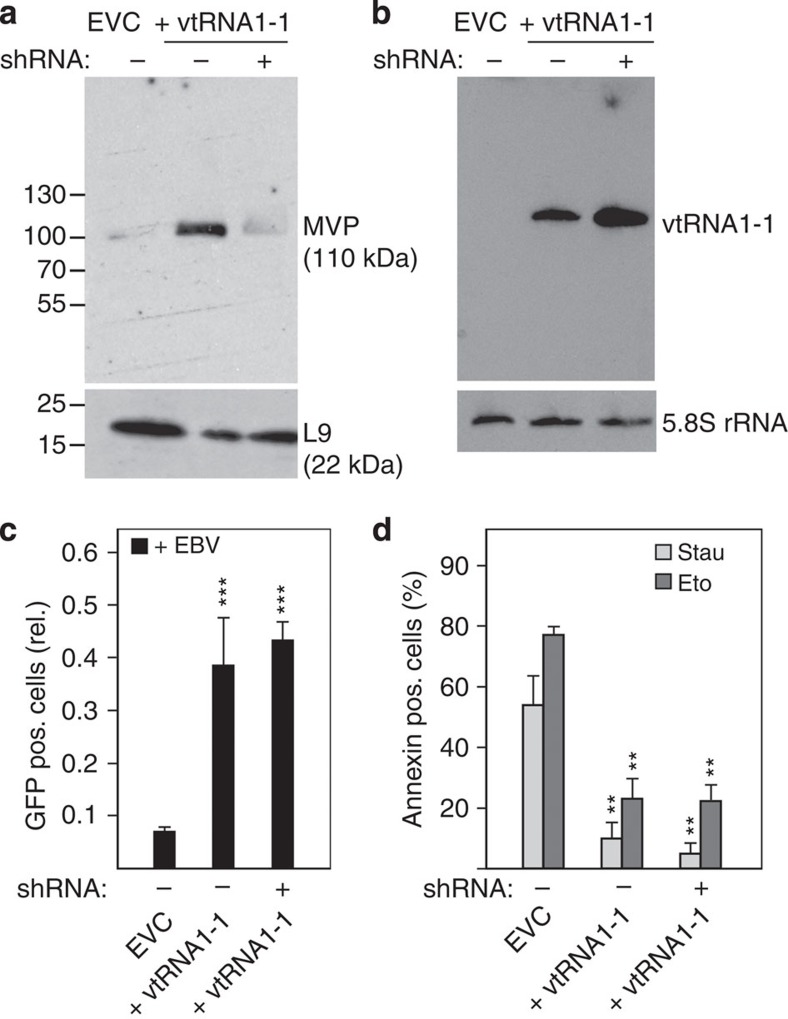
The vault complex does not contribute to the vtRNA1-1-mediated apoptosis resistance. (**a**) MVP levels in BL41 cells treated with the empty vector control (EVC) or ectopically expressing vtRNA1-1 in the absence (+vtRNA1-1) or presence of shRNA directed against the MVP were probed by western blot analyses. Ribosomal protein L9 served as loading control. Locations of molecular weight markers (kDa) are shown on the left. See also Supplementary Fig. 9. (**b**) The expression levels of vtRNA1-1 in the cells shown in **a** were assessed by northern blotting. The 5.8S rRNA served as internal loading control. (**c**) The effects of MVP knockdown on infection with EBV strain 2089 were monitored by fluorescence-activated cell sorting (FACS) analysis. The data originates from four individual experiments. (**d**) Apoptotic cells after staurosporine (Stau) or etoposide (Eto) treatment in BL41 cells expressing vtRNA1-1 in addition to high or low levels of the MVP were assessed by annexin V staining and were recorded on FL2 on a FACS device. The data represent the mean and standard deviation of three independent experiments. The amount of apoptotic cells in the untreated controls were <8% and were always subtracted from the staurosporine or etoposide treated cells. In **c** and **d**, significant differences relative (rel.) to the EVC were determined using the two-tailed unpaired Student’s *t*-test (***P<0.001, **P<0.01). pos., positive.

It has been demonstrated previously that only ∼5% of cellular vtRNA transcripts are actually associated with the vault complex while the remaining 95% remain free primarily in the cytoplasm or are located within significantly smaller and so far uncharacterized RNP particles^19^,^20^. Thus, it remained unclear whether or not vtRNA1-1 exerts its anti-apoptotic characteristic on its own or as part of the 13 MDa vault complex. To address this question, we specifically knocked down MVP, the principal protein component of the vault complex, in the vtRNA1-1 overexpressing BL41 cell line. To assess MVP assembled into genuine vault particles and not free protein, the western blots were performed on the pellet fraction of cell lysates that have passed a 100,000*g* centrifugation step. While the vtRNA1-1 overexpressing cell line had clearly elevated MVP levels compared with the parental BL41 cells as judged by western blot analysis, MVP could be almost quantitatively knocked down by the used shRNAs (Fig. 5a, Supplementary Fig. 9). At the same time, the ectopically expressed vtRNA1-1 levels remained high in the MVP knockdown line (Fig. 5b). Importantly, when this MVP knockdown cell line was subsequently infected with EBV for 72 h, virus establishment rates remained high (Fig. 5c). In addition, treatment with either staurosporine or etoposide did not alter the fraction of apoptotic cells (Fig. 5d), which is in contrast to what was observed after vtRNA1-1 knockdown (Fig. 3b). Hence, even though the BL41 cells had basically no MVP expression and consequently strongly reduced levels of genuine vault complexes, vtRNA1-1 expression was high and sufficient for triggering the anti-apoptotic effect. This finding provides further evidence that the anti-apoptotic effect of vtRNA1-1 is an intrinsic feature of this ncRNA and is independent of the vault complex.

### vtRNA1-1 affects the expression of central apoptosis regulators

**Figure 6:**
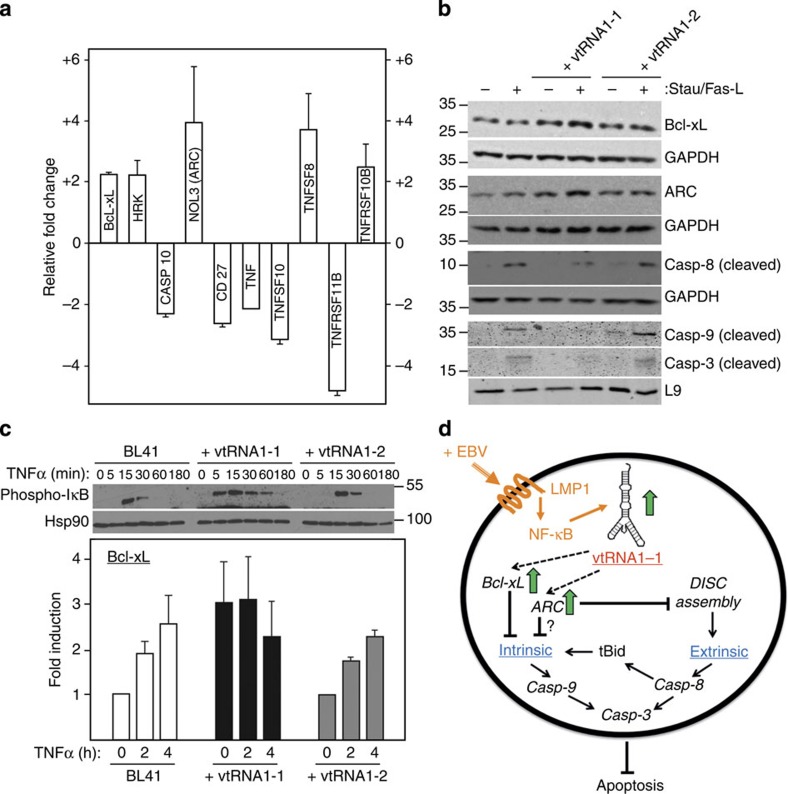
vtRNA1-1 modulates both the intrinsic and the extrinsic apoptosis pathway. (**a**) qPCR identifies 10 apoptosis marker genes. Only mRNAs with expression levels that differ more than 2-fold between BL41 and BL41 cells expressing vtRNA1-1 (after a 4.5 h staurosporine treatment) were taken into consideration. Upregulation is reflected by a positive and downregulation by a negative fold-change value. The data shown derive from averaging two biological replicates. (**b**) Protein levels of Bcl-xL, ARC and the cleaved caspases Casp-9 and Casp-3 in BL41 cells expressing vtRNA1-1 or vtRNA1-2 in the absence (−) or presence (+) of staurosporine (Stau) were assessed by western blot analyses. Cleavage of Casp-8 was monitored after Fas-L treatment. Proteins GAPDH or L9 served as loading controls. See also Supplementary Fig. 10. (**c**) Western blot analyses (upper panel) were used to monitor the kinetics and levels of phosphorylated IκB expression in BL41 cells, or in BL41 cells expressing vtRNA1-1 or vtRNA1-2, respectively, as a function of TNFα incubation (in minutes). Hsp90 served as loading control. The same cell lines were used to test for Bcl-xL mRNA expression by qPCR (lower panel). The data represent the mean and standard deviation of two independent experiments. Locations of molecular weight markers (kDa) are shown on the left (**b**) or right (**c**), respectively. (**d**) A putative model showing how vtRNA1-1 confers apoptosis resistance by modulating the intrinsic as well as the extrinsic pathways. When vtRNA1-1 levels are high (green arrow), such as in the presence of EBV infection and LMP1 signalling (orange), ARC and Bcl-xL become upregulated (green arrows). These proteins subsequently contribute to inhibiting the intrinsic as well as the extrinsic apoptotic pathway resulting in reduced levels of cleaved caspases 9 (intrinsic), 8 (extrinsic) and 3 (both pathways). In the latter case Bcl-xL neutralizes caspase-8 processed Bid at the outer mitochondrial membrane that links the extrinsic pathway to the mitochondrial pathway required for full effector caspase activation thus leading to resistance to Fas Ligand-induced cell death.

To investigate changes in cell death regulatory genes upon vtRNA1-1 expression within the extrinsic or intrinsic apoptotic pathways, a quantitative PCR array sampling 84 key marker genes involved in programmed cell death was performed. BL41 as well as BL41 cells overexpressing vtRNA1-1 were treated with staurosporine for 4.5 h (a time point at which Annexin V staining showed that BL41 started to commit to apoptosis) and total RNA was subsequently isolated and used for the qPCR array. By taking only apoptosis-related transcripts into consideration that reproducibly displayed an at least 2-fold change in abundance, resulted in a shortlist of 10 candidates (Fig. 6a; for the full list see Supplementary Table 1). Among those, NOL3, also known as ‘apoptotic protein with CARD’ (ARC), showed the highest upregulation upon vtRNA1-1 expression. This protein has been reported to act anti-apoptotic^34^,^35^ in both the extrinsic as well as the intrinsic pathway^36^. Another well-known apoptosis marker identified in our screen was Bcl-xL (also referred to as BCL2-like 1), which was upregulated in vtRNA1-1 expressing cells by a factor of 2.2 at the mRNA level (Fig. 6a). This pro-survival protein is located at the outer mitochondrial membrane and is involved in regulating the intrinsic apoptosis pathway but can also prevent extrinsic cell death in several cell types^37^. Another family of mRNAs that was deregulated upon vtRNA1-1 overexpression included the TNF/TNFR superfamily members TNF, TNFSF10 (TRAIL), TNFSF8 (CD30-L), TNFRSF11B (OPG) and TNFRSF10B (DR5, TRAIL-R2) (Fig. 6a). The latter result is more difficult to interpret in the context of apoptosis resistance, as some members of the TNFR superfamily can mediate extrinsic cell death as well as necroptosis but also participate in the inflammatory response upon viral infection^38^.

The qPCR array and cell death data indicate that vtRNA1-1 expression affects cell survival by modulating both the intrinsic (Fig. 2d, Fig. 3, Fig. 6a) as well as the extrinsic (Fig. 2e, Fig. 6a) apoptosis pathways presumably by controlling the function of common cell death regulators shared by these pathways. To substantiate these findings, western blot analyses of selected programmed cell death markers were performed. Indeed the anti-apoptotic proteins Bcl-xL and ARC were upregulated in BL41 cells expressing vtRNA1-1 upon staurosporine treatment, and this correlated with a lack of caspase activation (Fig. 6b; Supplementary Fig. 10). The vtRNA1-2 overexpression, on the other hand, failed to inhibit processing of caspases, indicative for their activation during apoptotic cell death.

To gain some mechanistic insight in vtRNA1-1-mediated apoptosis resistance, we treated BL41 cells expressing vtRNA1-1 or vtRNA1-2 as well as the parental BL41 (which lacks vtRNA expression) with the NF-κB agonist TNF. It became clear that in the presence of vtRNA1-1, basal pathway activity was already increased as indicated by the presence of phosphorylated IkB protein in unstimulated cells. In particular, the kinetics and amplitude of IkB phosphorylation, a key step preceding its degradation and subsequent NF-kB nuclear translocation, was significantly faster and maintained longer when compared with the parental cells expressing the vtRNA1-2 paralogue (Fig. 6c, Supplementary Fig. 10). This phenomenon was reflected in higher mRNA levels encoding for anti-apoptotic Bcl-xL that were not further augmented by TNF treatment (Fig. 6c) but correlated with a notable increase in Bcl-xL protein under baseline conditions (Fig. 6b).

In summary, we provide first evidence that the non-coding transcript vtRNA1-1 modulates the activity of both apoptosis inducing pathways by increasing levels of Bcl-xL, at least in part, by amplifying the baseline activity and potency of the NF-κB signaling cascade.

## Discussion

Despite the work of several research groups, the molecular function of the vtRNA or the vault complex remains elusive^39^,^40^. Here, we show that vtRNA1-1 is significantly upregulated upon expression of the EBV-encoded protein LMP1. Studying the possible causal link between EBV infection and vtRNA1-1 upregulation, we revealed that vtRNA1-1 triggers general apoptosis resistance in human Burkitt lymphoma cells, a phenotype which is independent of the vault RNP complex. Mutational analyses highlight the central domain of the vtRNA to contributing to this phenotype.

These data demonstrate that vtRNA1-1 possesses a cellular function in human cells, that is not connected to the vault complex. This is, however, not the first report that assigns a biological function to a vtRNA not connected to the vault complex *in vivo*. vtRNA1-1 was reported to serve as a precursor for miRNA-like regulatory small RNAs, named svRNAs^41^, that are able to dim CYP3A4 expression, a major drug metabolizing enzyme. However, in the presented work, we do not observe any smaller fragments of vtRNA1-1 on northern blots that might be related to the svRNAs described by Persson *et al*.^41^ Furthermore our mutational analyses point to the central domain of vtRNA1-1 as crucial determinant for the phenotype, while the previously described svRNAs originate from the terminal stem-loop. Another example for a vault complex-independent role of a vtRNA is the association of vtRNA2-1 (also known as pre-miR-886 or nc886) with the protein kinase R, which in consequence suppresses tumour cell growth^42^. Taken together, these recent reports and this study add to the growing body of evidence, that vtRNAs are functional ncRNA molecules on their own and fulfil cellular roles independently of the vault complex.

How is vtRNA1-1 expression linked to EBV infection and apoptosis resistance? In general, viruses target the most effective cellular switches to transform their hosts into virus factories^43^. NcRNAs appear to be a good target for virus factors due to their ability to rapidly regulate various critical cellular functions^44^. A recent study demonstrated that a long ncRNA (lncRNA) was specifically upregulated upon influenza A virus infection and that the abundance of this lncRNA determines infection rates^45^. The results by Winterling *et al*.^45^ and of the present work suggest that different viruses target specific ncRNAs of their hosts. Thus, particular ncRNAs, such as vtRNA1-1 in human B cells, can be regarded as key cellular switches required to transform host cells into viral factories. Our data suggest the following scenario: upon EBV infection, the viral gene product LMP1 activates, via its cytoplasmic C-terminal activator regions CTAR1 and CTAR2, the NF-κB pathway leading to vtRNA1-1 upregulation. EBV-triggered modulation of host transcription factors affecting polymerase III promoters has already been observed before and was suggested to stimulate cell growth^46^. LMP1 is known as the major oncogene of the Epstein–Barr virus and several studies suggest an anti-apoptotic and proliferative role of LMP1 (ref. 29). In another study, it was recently shown that LMP1 expression in nasopharyngeal carcinoma and lymphoblastoid cell lines elevates miRNA-21 levels, a known biomarker for chemo-resistance, likely via the PI3K/Akt/FOXO3a pathway^47^. This, in turn, downregulates the pro-apoptotic factors PDCD4 and Fas-L possibly contributing to chemoresistance. In human B cell lines, LMP1 functions differently and signals via the NF-κB pathway (Fig. 1e,f, Supplementary Figs 3c,d and 11). Our study identifies vtRNA1-1 as a downstream target of LMP1 that on its own appears sufficient to increase NF-κB signalling (Figs 1 and 6c). Among the affected NF-κB target genes is the key anti-apoptotic protein Bcl-xL. The central role vtRNA1-1 plays in this scenario is emphasized by the fact that Bcl-xL expression levels become independent of ligand-dependent NF-κB activation in BL41 cells ectopically expressing this ncRNA (Fig. 6c). These vtRNA1-1-specific effects (especially on Bcl-xL levels), in turn, protect cells from undergoing apoptosis. As a consequence, enhanced EBV establishment (Fig. 2b) and cell proliferation rates were observed (Supplementary Fig. 4a). The available data suggest that vtRNA1-1 expression leads to the modulation of both the extrinsic and the intrinsic apoptosis pathways (Figs 2d,e and 6a,b). Bcl-xL blocks both extrinsic as well as intrinsic apoptosis upstream of mitochondrial outer membrane permeabilization by preventing action of pro-apoptotic BH3-only proteins involved in intrinsic apoptosis, for example, Bim, Puma, required for staurosporine or etoposide killing^48^, as well as Bid, connecting the mitochondrial pathway to death receptor-mediated apoptosis in many cancer cells after caspase-8-mediated proteolysis^37^. Likewise ARC has been demonstrated to inhibit both apoptotic pathways through a direct interaction with Bax and/or Fas^49^. This is, to the best of our knowledge, the first report of an ncRNA, other than miRNAs, to directly affect both apoptotic pathways and thus is able to modulate programmed cell death in human cancer cells (Fig. 6d).

The vtRNAs received their names due to their association with the 13 MDa vault complex^7^. With its 96 copies, the major vault protein (MVP) is the dominant structural component of the vault complex. Even though MVP was upregulated due to vtRNA1-1 overexpression in BL41 cells, knockdown of MVP levels did not influence the anti-apoptotic effect of vtRNA1-1 (Fig. 5). This result suggests that the MVP, and thus the genuine vault complex, is not involved in the anti-apoptotic function of the vtRNA1-1. Recently it was suggested that the MVP is responsible for prolonged cell survival in human diploid fibroblasts (HDFs) in an age-dependent manner^14^. Senescent HDFs showed increased levels of MVP and resisted apoptotic stimuli. As a marked upregulation of vtRNA1-1 was also observed in senescent HDFs due to apoptotic stress, it cannot be ruled out that the observed apoptosis resistance phenotype actually originates from the vtRNA and not from the MVP. Also a more recent publication highlights the vtRNA1-1 levels and not the vault complex as a chemotherapy-resistance mediator^50^, thus emphasizing that vtRNAs can accomplish cellular functions even off the vault particle.

Although our study does not add to revealing the still elusive cell biology of the vault complex, it shows that ‘free’ vtRNA1-1 has an anti-apoptotic effect in human cancer cell lines. In that context, we note that >90% of vtRNA transcripts are not bound to the vault complex in human cells, thus the RNA nomenclature is misleading here since it implies a vault complex-related function. Future work needs to characterize the specific vtRNA1-1 RNP and the molecular mechanism that is responsible for the observed anti-apoptotic phenotype. As summarized in Fig. 6d, our data demonstrate a crucial role of vtRNA1-1, and particularly its central domain, in conferring general apoptosis resistance in different human cell lines (BL41, BL2, HS578T and HeLa) by modulating the expression of anti-apoptotic proteins.

## Methods

### Cell culture

Burkitt lymphoma (BL) was initially described as tumour of young children with a high EBV prevalence. BL cell lines have been established from patients, including the EBV-negative cell lines BL2 and BL41 (refs. 51, 52). IARC304 is an EBV-positive cell line derived from the same patient as BL2. The uniqueness of these cell lines and the relationship between BL2 and IARC304 has been confirmed by the German Collection of Microorganisms and Cell Cultures (DSMZ).The EBV-negative Burkitt lymphoma cell lines BL41, BL2, kindly provided by F. Grässer; Homburg/Saar, Germany and IARC304 were cultured in RPMI 1640 supplemented with 10% fetal calf serum, 292 μg ml^−1^ L-glutamin and antibiotics (100 U penicillin ml^−1^ and 100 μg streptomycin ml^−1^). After successful lentiviral transduction, positively infected cells were selected by treating them with 1 μg ml^−1^ puromycin (or 200 μg ml^−1^ G418) for at least 1 week. After ∼7 days of recovery time, the concentration of the antibiotic was raised to 2 μg ml^−1^ puromycin or 800 μg ml^−1^ G418, respectively. For experiments addressing NF-κB signaling, LMP1-expressing BL2 cells and HS578T cells were treated with 10 μM IKK Inhibitor VII (Calbiochem) for 18 h. HeLa cells were treated with 20 μM IKK Inhibitor VII (Calbiochem) for 18 h. Cells were collected by centrifugation, total RNA was prepared and vtRNA1-1 expression was analysed by northern blotting (see below). HeLa, HS578T and HEK 293 T cells (kindly provided by R. Berger, Department of Gynecology, Medical University Innsbruck, Austria) were grown in DMEM medium supplemented with 10% fetal calf serum, 292 μg ml^−1^ L-glutamine and antibiotics (100 U penicillin ml^−1^ and 100 μg streptomycin ml^−1^).

### Overexpression of EBV-encoded proteins

EBV-encoded proteins were amplified from plasmid-cloning vectors with specific PCR primers (Supplementary Table 2). The various LMP1 mutants are described in ref. 53. For the generation of the complete Gateway recombination sites, a further PCR was necessary (Supplementary Table2; AttB1 and AttB2). According to the Gateway Cloning Technology (Invitrogen), PCR products were recombined with a pDONOR-207 vector and subsequently sequence-verified entry clones were recombined with the lentiviral destination vector pHR-PGK-dest–SFFV-Puro. In the case of LMP2b and EBNA-LP, entry clones containing the CMV (for LMP2b) or the SV40 (for EBNA-LP) promoters were recombined with the lentiviral destination vector pHR-dest-SFFV-Puro.

### Lentiviral transduction

Lentiviral particles were generated in human HEK 293 T cells, which were transiently transfected with lentiviral plasmids containing cDNAs coding for all gene products mentioned in the text, together with the packaging plasmids pSPAX and the envelope plasmid pVSV-G. After 48 h and 72 h lentiviral supernatant was collected, sterile filtered (Whatman Puradisc FP30, 0.2 μM) and supplemented with polybrene (Millipore) to a final concentration of 4 μg ml^−1^ and added to the target cells overnight.

### Northern blot

Ten micrograms of total RNA (prepared with TRI Reagent (Sigma Aldrich) of ∼10 millions of cells according to the manufacturer’s protocol) isolated either from HeLa, HS578T cells or from the B cell lines BL41 and BL2 and derivatives thereof were separated on 8% denaturing polyacrylamide gels (7 M urea, 1 × TBE buffer), transferred onto nylon membranes, UV crosslinked and probed with 5′-[^32^P] end-labelled antisense DNA probes (Supplementary Table 2) as described^54^. Northern blot signals were visualized by autoradiography and quantified using ImageJ version 1.46. All quantified northern blot signals were normalized to the band intensities of the 5.8S or 5S rRNA loading controls.

### Western blot

Cells (except for MVP detection, see below) were solubilized with 2 × Lämmli buffer (4% SDS, 20% Glycerol, 125 mM Tris/HCl pH 6.8, 200 mM β-mercaptoethanol and bromphenol blue) or with NP40 lysis buffer (150 mM NaCl, 50 mM Hepes pH 7.5, 5 mM EDTA, 0.1% NP40, 1 mM PMSF). The same amount of protein content for each sample (determined by cell counting) was resolved by a 4–20% or 8–15% Tris/Glycin gradient SDS polyacrylamide gel, transferred onto a nitrocellulose membrane (Amersham Biosciences) and blocked with TBS containing Tween-20 in 5% nonfat dry milk overnight. The membranes were incubated with the primary antibodies at room temperature for 1 h. Horseradish peroxidase conjugated secondary antibodies or IRDye-conjugated secondary antibodies (LI-COR) were added for 1 h at room temperature. Immune complexes were visualized using an enhanced chemiluminescence (ECL) reagent, or ECL-femto (Pierce Thermo scientific) or were visualized with the Odyssey imaging system according to the manufacturer’s instructions. The following antibodies and dilutions for western blotting were used: mouse anti-MVP 1:500 (Santa Cruz); mouse anti-HA tag (16B12, Covance) 1:500; rabbit anti-FLAG 1:5,000 (F7425, Sigma), Histidine Tag (6xHis) mouse anti-histidine Tag monoclonal Antibody (clone 3D5, Life Technologies); rat anti-EBNA1 1:50 (ref. 55); rat anti-EBNA2 (1:20, ref. 56); mouse anti-EBV 1:1,000 (Dianova),rabbit anti–PDCD4 1:1,000 (D29C6, Cell Signaling); rabbit anti-cleaved caspase-3 1:1,000 (5A1E, Cell Signaling); rabbit anti-cleaved caspase-9 1:1,000 (D2D4, Cell Signaling); mouse anti-cleaved caspase-8 1:1,000 (11G10, Cell Signaling); goat anti-mouse IgG HRP Conjugate 1:10,000 (Invitrogen); goat anti–rabbit IgG HRP Conjugate 1:15,000 (Pierce); anti-ribosomal protein L9 (Santa Cruz); rabbit anti-Bcl-xL 1:1,000 (54H6, Cell Signaling), rabbit anti-ARC 1:1,000 (sc-11435, Santa Cruz); rabbit anti-GAPDH 1:1,000 (14C10, Cell Signaling), rabbit anti-NFκB p65 (Santa Cruz, sc-372) 1: 500, rabbit anti Phospho-IkBα (Ser32) 1:250 (14D4, Cell Signaling), mouse anti-HSP 90 1:1,000 (AC-16, Santa Cruz).

For MVP detection, a concentration step namely a P100 preparation was necessary. 5 × 10^6^ cells were washed with phosphate-buffered saline (PBS), pelleted by centrifugation (450*g* for 5 min), snap frozen to disrupt the cell membranes, dissolved in 1 ml of buffer A complete (50 mM Tris-HCl pH7.4, 1.5 mM MgCl_2_, 75 mM NaCl, 1% Triton X-100, 1 mM DTT, 1 mM PMSF, 0.1% Nonidet–P40, protease inhibitor cocktail complete Mini EDTA-free 1 × (Roche)) followed by incubation for 10 min on ice and subsequent centrifugation at 20,000*g* at 4 °C for 20 min. The resulting supernatant was again centrifuged in an ultracentrifuge for 1 h at 100,000*g* (TLA-55 rotor). The supernatant was discarded and the pellet, containing vault complexes, was dissolved in 60 μl of buffer A complete. Then, 60 μl of 2 × Laemmli sample buffer (without β-mercaptoethanol but including 7 M urea) was added followed by vigorous vortexing, sonication (for 5 sec at 200 W with a BRANSON SONIFIER 150) and supplemented with 50 mM β-mercaptoethanol (final concentration).

### Real-time PCR

Total RNA was isolated from different BL2 and BL41 cell lines with TRI Reagent (Sigma Aldrich) according to the manufacturer’s protocol. An amount of 2.5 μg of total RNA was reverse transcribed to cDNA using Superscript II (Invitrogen Karlsruhe) and random hexamer primers following the manufacturer’s instructions. The expression of vtRNA1-1 and TATA box binding protein (TBP) mRNA was investigated by real-time PCR using Taq Man primers and probes (ABI). PCR was performed using Rotor Gene 6200 real-time thermocycler with the following thermal conditions: 50 °C for 2 min, 95 °C for 10 min, 40 cycles each of 95° for 15 s and 60 °C for 1 min. Reactions were performed in triplicates. For the NF-kB target genes (Bcl-xL, IL6, c-Flip) the Brilliant III ultra-fast SYBR Green qPCR master mix (Agilent Technologies) was used on the Rotor gene 6200 thermocycler with the following conditions: 95 °C for 3 min, 40 cycles each of 95 °C for 10 s and 60 °C for 15 s. Quantification was calculated using the ^ΔΔ^CT method with TBP mRNA as endogenous control. The used primers are listed in Supplementary Table 2.

For transcriptionally regulated target search a RT^2^ Profiler PCR Array (Qiagen) was performed. A total 2.5 × 10^6^ BL41 as well as BL41 vtRNA1-1 expressing cells were incubated with staurosporine for 4.5 h. The cells were pelleted by centrifugation (400*g*, 5 min) and RNA was extracted according to the TRI-Reagent (Sigma Aldrich) protocol. A total 8 μg of the extracted RNA was treated with 1 U of RQ DNase (Promega) in 1 × RQ DNase buffer for 30 min at 37 °C to get rid of residual DNA. Afterwards the RNA was purified with the RNeasy Mini Kit according to Qiagen’s protocol and reverse transcribed into cDNA using the RT^2^ First Strand Kit as described in the RT^2^ Profiler PCR Array Handbook (12/2012). The array was run on an ABI ViiA 7 cycler. Cycling conditions as well as data analysis was performed according to the manufacturer’s recommendations. Expression levels of the different genes were normalized to the average of five different housekeeping genes (as described in the RT^2^ Profiler PCR Array Handbook) and ‘fold changes’ were calculated using the comparative 2^−(ΔΔCT)^ method where BL41 served as calibrator and BL41 expressing vtRNA1-1 as target.

### Chromatin immunoprecipitation assay

A total 1 × 10^7^ BL2 cells were exposed to 1% formaldehyde at room temperature for 10 min to obtain protein-DNA cross linking. After sonification for 15 min (40% output, 30 sec on/off), the lysates were centrifuged at 13,000*g*, 15 min, 4 °C. The supernatants were diluted to 250 μg ml^−1^ in ChiP dilution buffer (25 mM HEPES/pH 7.5, 140 mM NaCl, 1 mM EDTA/pH 8.0, 0.5 mM EGTA/pH 8.0). Five microgram of anti-NF-κB p65 antibody (C20, sc-372X; Santa Cruz Biotechnology) or 5 μg of control IgG were coupled to protein A magnet beads (Dynabeads; Life Technology) for 8 h at 4 °C. Subsequently, the bead-coupled antibodies were washed three times in 1 × PBS, 0.5% bovine serum albumin (BSA) and incubated with 250 μg ml^−1^ sonified chromatin overnight at 4 °C. The protein-DNA-antibody complexes were washed five times with cold wash buffer (50 mM HEPES-KOH/pH7.5, 500 mM LiCl, 1 mM EDTA, 1% NP-40, 0.7% Na-deoxycholate), once with TE+50 mM NaCl and resuspended in elution buffer (50 mM Tris-HCl/pH 8.0, 10 mM EDTA, 1% SDS). Crosslinks were reversed in elution buffer for 9 h at 65 °C. After RNase A and proteinase K treatment, the DNA was purified by phenol/chloroform extraction and ethanol precipitated. Quantitative real-time PCR (3 min 95 °C, 40 cycles each 10 s 95 °C and 15 s 50 °C) was performed with Brilliant III ultra-fast SYBR Green qPCR master mix (Agilent Technologies) to amplify a distinct region of the vtRNA1-1 promoter and of the vtRNA1-2 promoter region. Primer sequences are listed in Supplementary Table 2. Quantification was calculated as fold-enrichment, representing the ChiP signal as x-fold increase in signal to the no-antibody (mock IgG) signal.

### Annexin V staining

After treatment with 50 nM (f.c.) staurosporine for 24 h, 850 nM (f.c.) etoposide for 24 h, 500 ng Fas antibody clone CH11 (Millipore) for 48 h, or 2089 EBV (at a multiplicity of infection of 5) for 72 h, ∼2 × 10^5^ of suspended cells were collected by centrifugation (450*g*, 5 min), then washed once with ice-cold PBS, again centrifuged and finally dissolved in 100 μl 1 × Annexin binding buffer (0.01 M Hepes pH 7.4, 0.15 M NaCl, 2.5 mM CaCl_2_). Annexin V PE (2.5 μl) was added to the cells, which were then incubated for 15 min in the dark. The samples were analysed by flow cytometry within 1 h on a FACSCalibur (Becton Dickinson, Schwechat, Austria) and gated on the basis of forward versus side scatter for size. The number of apoptotic cells was determined by recording the FL-2 channel.

### MVP knockdown

A constitutive MVP knockdown cell line was constructed using a lentiviral shRNA expression vector containing a neomycin resistance gene for selection of positive clones. The target sequences for the performed knockdown was the following: 5′-CCCATACCACTATATCCATGT-3′. A plasmid expressing the shRNA targeting MVP was generated by annealing phosphorylated oligonucleotides, followed by ligation into a BglII/HindIII digested pENTR-THT vector. Thereafter, a sequence-verified clone was used for L/R recombination into the final lentiviral destination vector thereby generating pHR-THT-MVP shRNA-SFFV-Neo. The whole shDNA sequence for MVP knockdown is listed in Supplementary Table 2.

### Vault RNA expression

vtRNA1-1 with its upstream and downstream regulatory regions was amplified from genomic DNA of BL2/B95.8 cells within two PCR reactions. The specific amplification of the region of vtRNA1-1 was done using a nested PCR with the 5′hvg1-big and 3’hvg1-big primers. The product of the nested PCR (4.128 kb) was purified via agarose gel electrophoresis and it served as a template for another PCR resulting in a 644-nt long PCR product (hvg1-2 as well as hvg 1-4 primer containing an EcoRI and BamHI restriction site, respectively and all necessary regulatory regions in the vtRNA1-1 gene surrounding). The PCR product was then agarose gel purified, digested with EcoRI and BamHI restriction enzymes and cloned into a promoterless entry vector. Using Invitrogen’s Gateway recombination system, a sequence-verified entry clone was recombined with the lentiviral destination vector pHR-dest-SFFV-Puro resulting in pHR-vtRNA1-1_locus-SFFV-Puro. The vtRNA1-2 as well as the vtRNA1-3 gene locus encompassing upstream and downstream regulatory regions was chemically synthesized (Life Technologies, Geneart), digested with XhoI and EcoRI and cloned into pENTR-U243. Five different vtRNA1-1 mutations were generated via overlapping PCR. The listed primers (Supplementary Table 2) were used to generate vtRNA1-1 mutants M1 to M5 from the pENTR-U243-vtRNA1-1 plasmid.

### vtRNA1-1 knockdown

A constitutive vtRNA1-1 knockdown cell line was constructed analogous to the described MVP knockdown cell lines (see above). The target sequence on vtRNA1-1 was the following: 5′-GGCUGGCUUUAGCUCAGCG-3′. The whole shDNA sequence for the vtRNA1-1 knockdown is listed in Supplementary Table 2.

To reduce endogenous vtRNA1-1 levels in HeLa cells or HS578T cells, chemically modified chimeric ASOs were used. Therefore 5 × 10^6^ HeLa cells were transiently transfected (jetPEI, Polyplus) with 1 μM ASOs (Exiqon), directed against vtRNA1-1 or as a control against vtRNA1-2. After 24 h, cells were collected by centrifugation (450*g*, 5 min), stained with Annexin V and analysed by flow cytometry (as described above). ASOs were designed as RNA/DNA/RNA chimeric oligonucleotides with a phosphorothioate backbone. Ten central deoxyribonucleotides are flanked by five 2′-O-methyl modified ribonucleotides on both sides (Supplementary Table 2).

## Additional information

**How to cite this article:** M Amort *et al*. Expression of the vault RNA protects cells from undergoing apoptosis. *Nat. Commun*. 6:7030 doi: 10.1038/ncomms8030 (2015).

## Supplementary Material

Supplementary Information Supplementary Figures 1-11, Supplementary Tables 1-2 and Supplementary References

## References

[b1] Huttenhofer A., Schattner P. & Polacek N. Non-coding RNAs: hope or hype? Trends Genet. 21, 289–297 (2005). 15851066 10.1016/j.tig.2005.03.007

[b2] Amaral P. P., Dinger M. E., Mercer T. R. & Mattick J. S. The eukaryotic genome as an RNA machine. Science 319, 1787–1789 (2008). 18369136 10.1126/science.1155472

[b3] Gebetsberger J. & Polacek N. Slicing tRNAs to boost functional ncRNA diversity. RNA Biol. 10, 1798–1806 (2013). 24351723 10.4161/rna.27177 PMC3917982

[b4] Sabin L. R., Delas M. J. & Hannon G. J. Dogma derailed: the many influences of RNA on the genome. Mol. Cell 49, 783–794 (2013). 23473599 10.1016/j.molcel.2013.02.010 PMC3825098

[b5] Tuck A. C. & Tollervey D. RNA in pieces. Trends Genet. 27, 422–432 (2011). 21741109 10.1016/j.tig.2011.06.001

[b6] Mattick J. S. RNA regulation: a new genetics? Nat. Rev. 5, 316–323 (2004). 10.1038/nrg1321 15131654

[b7] Kedersha N. L. & Rome L. H. Isolation and characterization of a novel ribonucleoprotein particle: large structures contain a single species of small RNA. J. Cell Biol. 103, 699–709 (1986). 2943744 10.1083/jcb.103.3.699 PMC2114306

[b8] Stadler P. F. . Evolution of vault RNAs. Mol. Biol. Evol. 26, 1975–1991 (2009). 19491402 10.1093/molbev/msp112

[b9] van Zon A. . Vault mobility depends in part on microtubules and vaults can be recruited to the nuclear envelope. Exp. Cell Res. 312, 245–255 (2006). 16310186 10.1016/j.yexcr.2005.10.016

[b10] Kickhoefer V. A. . Vaults are up-regulated in multidrug-resistant cancer cell lines. J. Biol. Chem. 273, 8971–8974 (1998). 9535882 10.1074/jbc.273.15.8971

[b11] Kitazono M. . Multidrug resistance and the lung resistance-related protein in human colon carcinoma SW-620 cells. J. Nat. Cancer Inst. 91, 1647–1653 (1999). 10511592 10.1093/jnci/91.19.1647

[b12] Chung J. H., Ginn-Pease M. E. & Eng C. Phosphatase and tensin homologue deleted on chromosome 10 (PTEN) has nuclear localization signal-like sequences for nuclear import mediated by major vault protein. Cancer Res. 65, 4108–4116 (2005). 15899801 10.1158/0008-5472.CAN-05-0124

[b13] Kim E. . Crosstalk between Src and major vault protein in epidermal growth factor-dependent cell signalling. FEBS J. 273, 793–804 (2006). 16441665 10.1111/j.1742-4658.2006.05112.x

[b14] Ryu S. J. . On the role of major vault protein in the resistance of senescent human diploid fibroblasts to apoptosis. Cell Death Differ. 15, 1673–1680 (2008). 18600231 10.1038/cdd.2008.96

[b15] Kowalski M. P. . Host resistance to lung infection mediated by major vault protein in epithelial cells. Science 317, 130–132 (2007). 17615361 10.1126/science.1142311 PMC3685177

[b16] Shimamoto Y. . Direct activation of the human major vault protein gene by DNA-damaging agents. Oncol. Rep. 15, 645–652 (2006). 16465425

[b17] Vollmar F. . Assembly of nuclear pore complexes mediated by major vault protein. J. Cell Sci. 122, 780–786 (2009). 19240118 10.1242/jcs.039529

[b18] Stephen A. G. . Assembly of vault-like particles in insect cells expressing only the major vault protein. J. Biol. Chem. 276, 23217–23220 (2001). 11349122 10.1074/jbc.C100226200

[b19] Nandy C. . Epstein-barr virus-induced expression of a novel human vault RNA. J. Mol. Biol. 388, 776–784 (2009). 19298825 10.1016/j.jmb.2009.03.031

[b20] Kong L. B., Siva A. C., Kickhoefer V. A., Rome L. H. & Stewart P. L. RNA location and modeling of a WD40 repeat domain within the vault. RNA 6, 890–900 (2000). 10864046 10.1017/s1355838200000157 PMC1369965

[b21] Abbondanza C. . Interaction of vault particles with estrogen receptor in the MCF-7 breast cancer cell. J. Cell Biol. 141, 1301–1310 (1998). 9628887 10.1083/jcb.141.6.1301 PMC2132791

[b22] Mrazek J., Kreutmayer S. B., Grasser F. A., Polacek N. & Huttenhofer A. Subtractive hybridization identifies novel differentially expressed ncRNA species in EBV-infected human B cells. Nucleic Acids Res. 35, e73 (2007). 17478510 10.1093/nar/gkm244 PMC1904266

[b23] Shah K. M. & Young L. S. Epstein-Barr virus and carcinogenesis: beyond Burkitt's lymphoma. Clin. Microbiol. Infect. 15, 982–988 (2009). 19874382 10.1111/j.1469-0691.2009.03033.x

[b24] Thorley-Lawson D. A. Epstein-Barr virus: exploiting the immune system. Nat. Rev. Immunol. 1, 75–82 (2001). 11905817 10.1038/35095584

[b25] Abdulkarim B. . Antiviral agent cidofovir decreases Epstein-Barr virus (EBV) oncoproteins and enhances the radiosensitivity in EBV-related malignancies. Oncogene 22, 2260–2271 (2003). 12700662 10.1038/sj.onc.1206402

[b26] Murray R. J. . Epstein-Barr virus-specific cytotoxic T-cell recognition of transfectants expressing the virus-coded latent membrane protein LMP. J. Virol. 62, 3747–3755 (1988). 2843672 10.1128/jvi.62.10.3747-3755.1988 PMC253518

[b27] Bain M., Watson R. J., Farrell P. J. & Allday M. J. Epstein-Barr virus nuclear antigen 3C is a powerful repressor of transcription when tethered to DNA. J. Virol. 70, 2481–2489 (1996). 8642676 10.1128/jvi.70.4.2481-2489.1996 PMC190092

[b28] Cludts I. & Farrell P. J. Multiple functions within the Epstein-Barr virus EBNA-3A protein. J. Virol. 72, 1862–1869 (1998). 9499037 10.1128/jvi.72.3.1862-1869.1998 PMC109476

[b29] Kieser A. Signal transduction by the Epstein-Barr virus oncogene latent membrane protein 1 (LMP1). Signal Transduct. 7, 20–33 (2007).

[b30] Wong D. . Extensive characterization of NF-kappaB binding uncovers non-canonical motifs and advances the interpretation of genetic functional traits. Genome Biol. 12, R70 (2011). 21801342 10.1186/gb-2011-12-7-r70 PMC3218832

[b31] The ENCODE project consortium. A user's guide to the encyclopedia of DNA elements (ENCODE). PLoS Biol. 9, e1001046 (2011). 21526222 10.1371/journal.pbio.1001046 PMC3079585

[b32] Delecluse H. J., Hilsendegen T., Pich D., Zeidler R. & Hammerschmidt W. Propagation and recovery of intact, infectious Epstein-Barr virus from prokaryotic to human cells. Proc. Natl Acad. Sci. USA 95, 8245–8250 (1998). 9653172 10.1073/pnas.95.14.8245 PMC20961

[b33] Ideue T., Hino K., Kitao S., Yokoi T. & Hirose T. Efficient oligonucleotide-mediated degradation of nuclear noncoding RNAs in mammalian cultured cells. RNA 15, 1578–1587 (2009). 19535462 10.1261/rna.1657609 PMC2714749

[b34] Koseki T., Inohara N., Chen S. & Nunez G. ARC, an inhibitor of apoptosis expressed in skeletal muscle and heart that interacts selectively with caspases. Proc. Natl Acad. Sci. USA 95, 5156–5160 (1998). 9560245 10.1073/pnas.95.9.5156 PMC20230

[b35] Wang M., Qanungo S., Crow M. T., Watanabe M. & Nieminen A. L. Apoptosis repressor with caspase recruitment domain (ARC) is expressed in cancer cells and localizes to nuclei. FEBS Lett. 579, 2411–2415 (2005). 15848180 10.1016/j.febslet.2005.03.040

[b36] Ludwig-Galezowska A. H., Flanagan L. & Rehm M. Apoptosis repressor with caspase recruitment domain, a multifunctional modulator of cell death. J. Cell. Mol. Med. 15, 1044–1053 (2011). 21129150 10.1111/j.1582-4934.2010.01221.x PMC3822617

[b37] Czabotar P. E., Lessene G., Strasser A. & Adams J. M. Control of apoptosis by the BCL-2 protein family: implications for physiology and therapy. Nat. Rev. Mol. Cell Biol. 15, 49–63 (2014). 24355989 10.1038/nrm3722

[b38] Silke J. & Hartland E. L. Masters, marionettes and modulators: intersection of pathogen virulence factors and mammalian death receptor signaling. Curr. Opin. Immunol. 25, 436–440 (2013). 23800628 10.1016/j.coi.2013.05.011

[b39] Berger W., Elbling L. & Micksche M. Expression of the major vault protein LRP in human non-small-cell lung cancer cells: activation by short-term exposure to antineoplastic drugs. Int. J. Cancer 88, 293–300 (2000). 11004683

[b40] Lara P. C., Pruschy M., Zimmermann M. & Henriquez-Hernandez L. A. MVP and vaults: a role in the radiation response. Radiat. Oncol. 6, 148 (2011). 22040803 10.1186/1748-717X-6-148 PMC3216873

[b41] Persson H. . The non-coding RNA of the multidrug resistance-linked vault particle encodes multiple regulatory small RNAs. Nat. Cell Biol. 11, 1268–1271 (2009). 19749744 10.1038/ncb1972

[b42] Lee K. S. . nc886, a non-coding RNA of anti-proliferative role, is suppressed by CpG DNA methylation in human gastric cancer. Oncotarget 5, 3944–3955 (2014). 25003254 10.18632/oncotarget.2047 PMC4116533

[b43] Novoa R. R. . Virus factories: associations of cell organelles for viral replication and morphogenesis. Biol. Cell 97, 147–172 (2005). 15656780 10.1042/BC20040058 PMC7161905

[b44] Huang Y. . Molecular functions of small regulatory noncoding RNA. Biochemistry (Mosc) 78, 221–230 (2013). 23586714 10.1134/S0006297913030024

[b45] Winterling C. . Evidence for a crucial role of a host non-coding RNA in influenza A virus replication. RNA Biol. 11, 66–75 (2014). 24440876 10.4161/rna.27504 PMC3929426

[b46] Felton-Edkins Z. A. . Epstein-Barr virus induces cellular transcription factors to allow active expression of EBER genes by RNA polymerase III. J. Biol. Chem. 281, 33871–33880 (2006). 16956891 10.1074/jbc.M600468200

[b47] Yang G. D. . Epstein-Barr Virus_Encoded LMP1 upregulates microRNA-21 to promote the resistance of nasopharyngeal carcinoma cells to cisplatin-induced Apoptosis by suppressing PDCD4 and Fas-L. PLoS ONE 8, e78355 (2013). 24194922 10.1371/journal.pone.0078355 PMC3806812

[b48] Erlacher M. . Puma cooperates with Bim, the rate-limiting BH3-only protein in cell death during lymphocyte development, in apoptosis induction. J. Exp. Med. 203, 2939–2951 (2006). 17178918 10.1084/jem.20061552 PMC2118188

[b49] Danial N. N. & Korsmeyer S. J. Cell death: critical control points. Cell 116, 205–219 (2004). 14744432 10.1016/s0092-8674(04)00046-7

[b50] Gopinath S. C., Wadhwa R. & Kumar P. K. Expression of noncoding vault RNA in human malignant cells and its importance in mitoxantrone resistance. Mol. Cancer Res. 8, 1536–1546 (2010). 20881010 10.1158/1541-7786.MCR-10-0242

[b51] Cohen J. H. . B-cell maturation stages of Burkitt's lymphoma cell lines according to Epstein-Barr virus status and type of chromosome translocation. J. Nat. Cancer Inst. 78, 235–242 (1987). 3027441

[b52] Lenoir G. M. . The use of lymphomatous and lymphoblastoid cell lines in the study of Burkitt’s lymphoma. IARC Sci. Publ. 60, 309–318 (1985). 3934070

[b53] Shkoda A. . The germinal center kinase TNIK is required for canonical NF-kappaB and JNK signaling in B-cells by the EBV oncoprotein LMP1 and the CD40 receptor. PLoS Biol. 10, e1001376 (2012). 22904686 10.1371/journal.pbio.1001376 PMC3419181

[b54] Gebetsberger J., Zywicki M., Kunzi A. & Polacek N. tRNA-derived fragments target the ribosome and function as regulatory non-coding RNA in Haloferax volcanii. Archaea 2012, 260909 (2012). 23326205 10.1155/2012/260909 PMC3544259

[b55] Grasser F. A. . Monoclonal antibodies directed against the Epstein-Barr virus-encoded nuclear antigen 1 (EBNA1): immunohistologic detection of EBNA1 in the malignant cells of Hodgkin's disease. Blood 84, 3792–3798 (1994). 7949135

[b56] Zimber-Strobl U. . The Epstein-Barr virus nuclear antigen 2 interacts with an EBNA2 responsive cis-element of the terminal protein 1 gene promoter. EMBO J. 12, 167–175 (1993). 8381349 10.1002/j.1460-2075.1993.tb05642.x PMC413188

