# Supplementary material for: Expression of the vault RNA protects cells from undergoing apoptosis
Source: Nat Commun. 2015 May 8;6:7030. doi: 10.1038/ncomms8030 (PMC4430821; doi:10.1038/ncomms8030)
Supplement: Supplementary Information — Supplementary Figures 1-11, Supplementary Tables 1-2 and Supplementary References [file ncomms8030-s1.pdf]

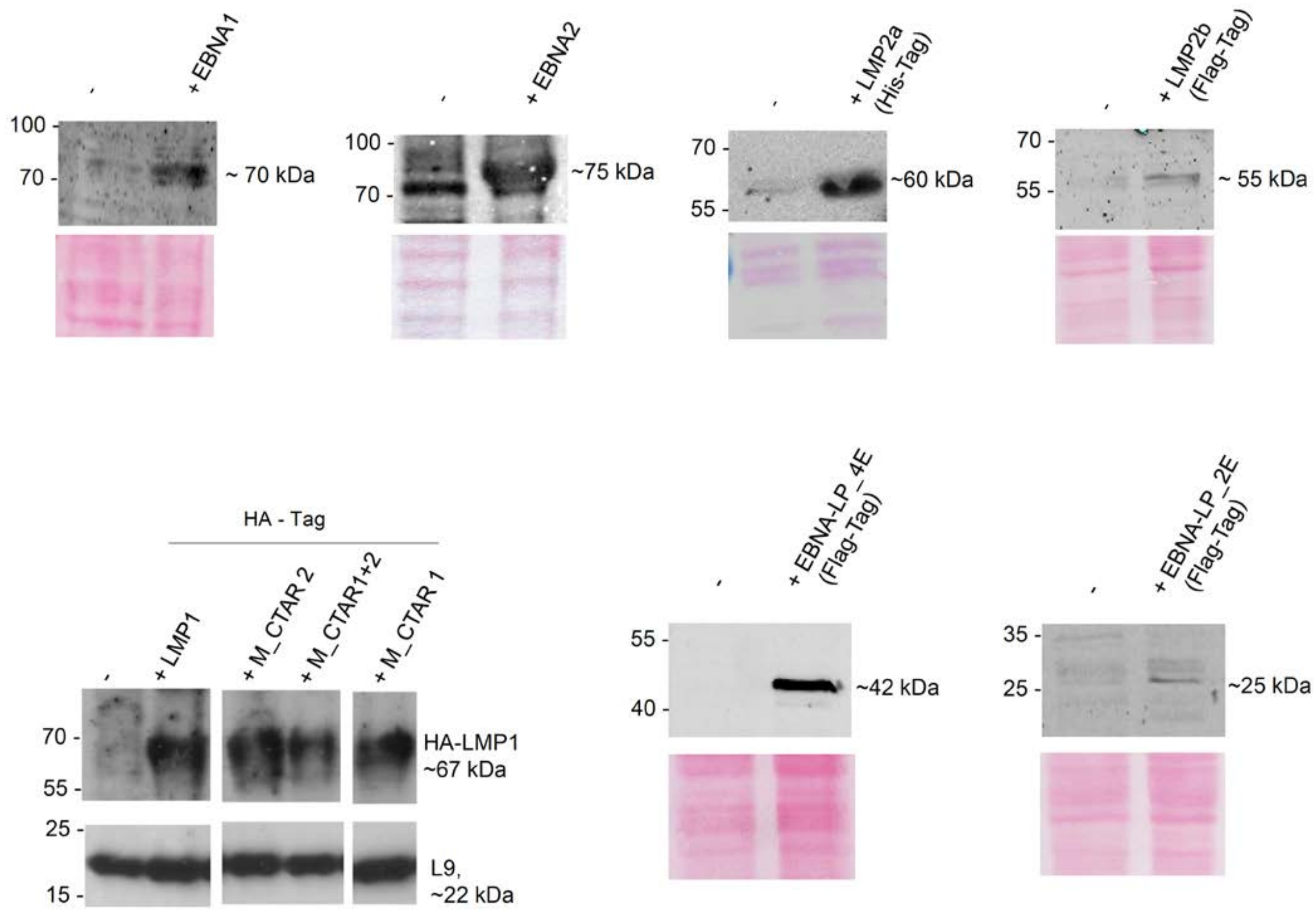


**Supplementary Figure 1: Overexpression of EBV-encoded proteins**

Western blot analysis of the expression levels of EBV-encoded latency III proteins in BL2 cells. The Ponceau S staining of the membranes or the western blot signal for ribosomal protein L9 served as loading controls. The size of the proteins is marked on the right. (-) indicates untreated BL2 cells without any overexpression; LMP2a was tagged with a His-Tag, LMP2b and EBNA_LP (2E and 4E) with a FLAG-Tag, and LMP1 as well as all LMP1-mutants with an HA-Tag and were detected with specific anti-tag antibodies. Positions of molecular weight markers (kDa) are indicated on the left.

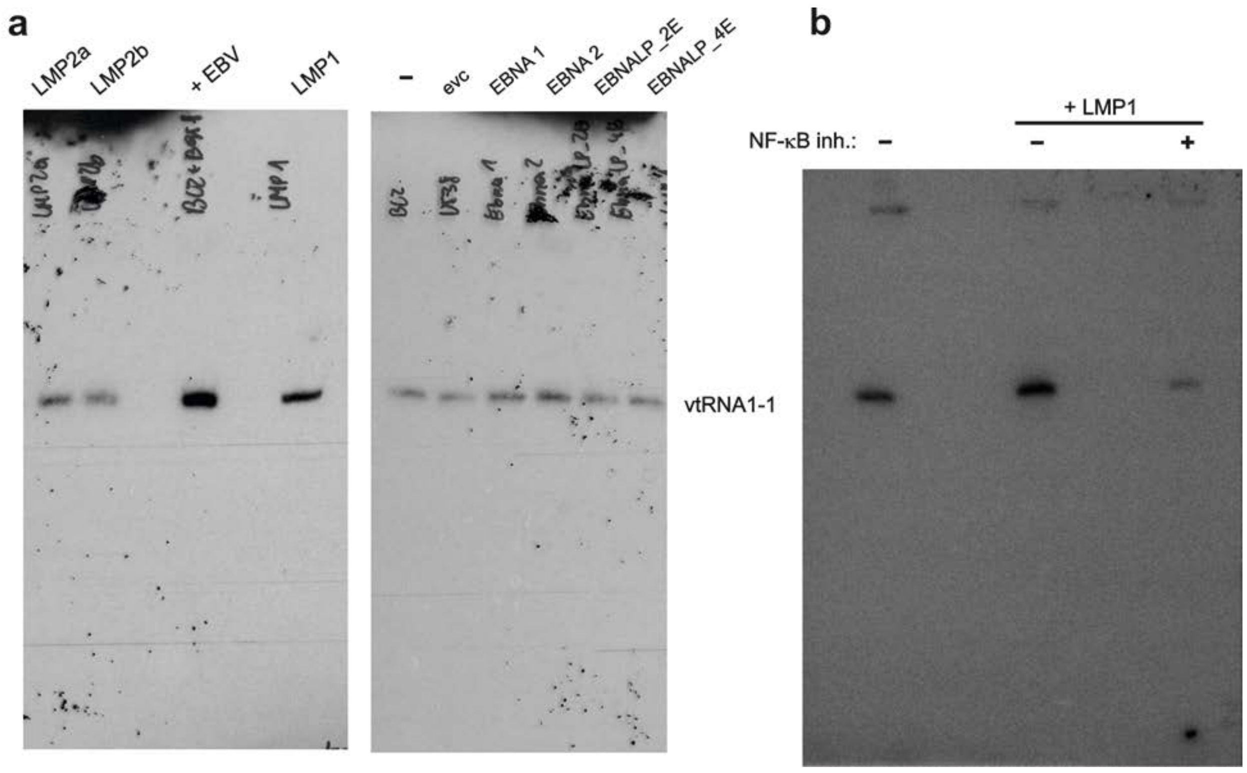


**Supplementary Figure 2: Uncropped northern blots of vtRNA levels in response to expression of EBV-encoded latency III proteins. (a)** Northern blot analysis was employed to assess the effects of overexpressing EBV-encoded proteins on the vtRNA1-1 levels in different BL2 cell lines. (-) indicate untreated BL2 cells, whereas (evc) indicates the empty vector control. EBV strain B95.8 was used as positive infection control (+ EBV). **(b)** In the presence of an NF-κB inhibitor northern blot analysis showed no LMP1-dependent up-regulation of vtRNA1-1.

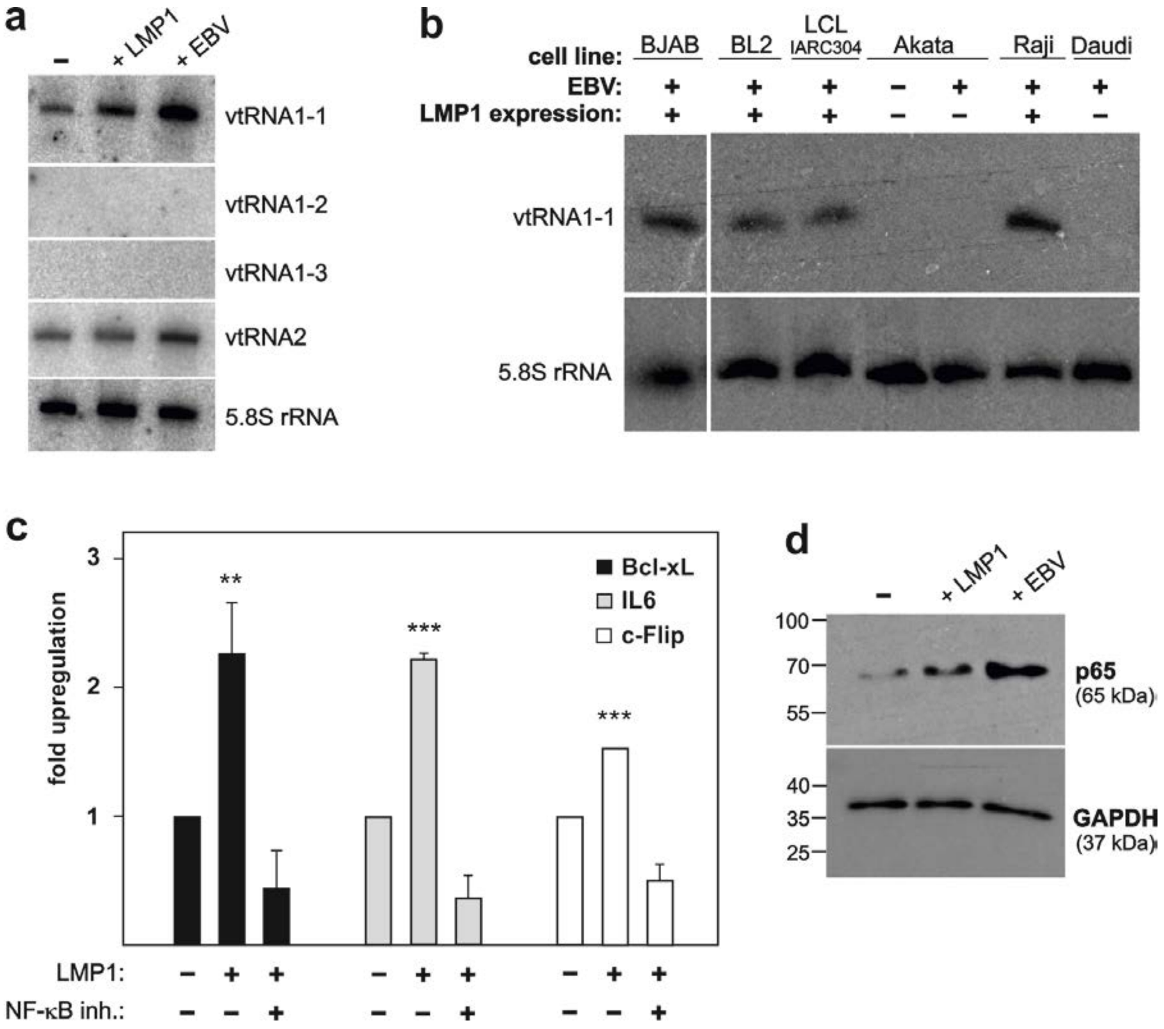


**Supplementary Figure 3: LMP1 expression in BL2 cells and its effects on vtRNA levels and NF-κB signaling**

**(a)** A northern blot analyses showing that LMP1 expression does neither affect vtRNA1-2, vtRNA1-3, nor vtRNA2 levels. As a positive control, vtRNA levels in EBV infected BL2 cells (+EBV) are shown. **(b)** The vtRNA1-1 levels in different B cell lines (either EBV infected or not) were assessed by northern blot analysis. Only in cell lines known to express the EBV-encoded LMP1 (ref [1]) signals for the vtRNA1-1 were evident. In (a) and (b) 5.8S rRNA served as internal loading control. **(c)** Real time qPCR of known NF-κB target genes Bcl-xL, IL6 and c-Flip was monitored in untreated BL2 cells (-) and in LMP1 expressing cells and compared to cells treated with the NF-κB inhibitor. 'Fold induction' was calculated using the comparative Δct method where BL2 served as calibrator and TBP as housekeeping gene. Data shown are the mean values and standard deviations from three experiments. Significant differences in fold induction relative to the untreated BL2 cells were determined using the two-tailed unpaired Student's t-test (*** *P*<0.001, ** *P*<0.01). **(d)** Western Blot analysis of NF-κB p65 in different BL2 cell lines. GAPDH serves as internal loading control. Positions of molecular weight markers (in kDa) are indicated on the left.

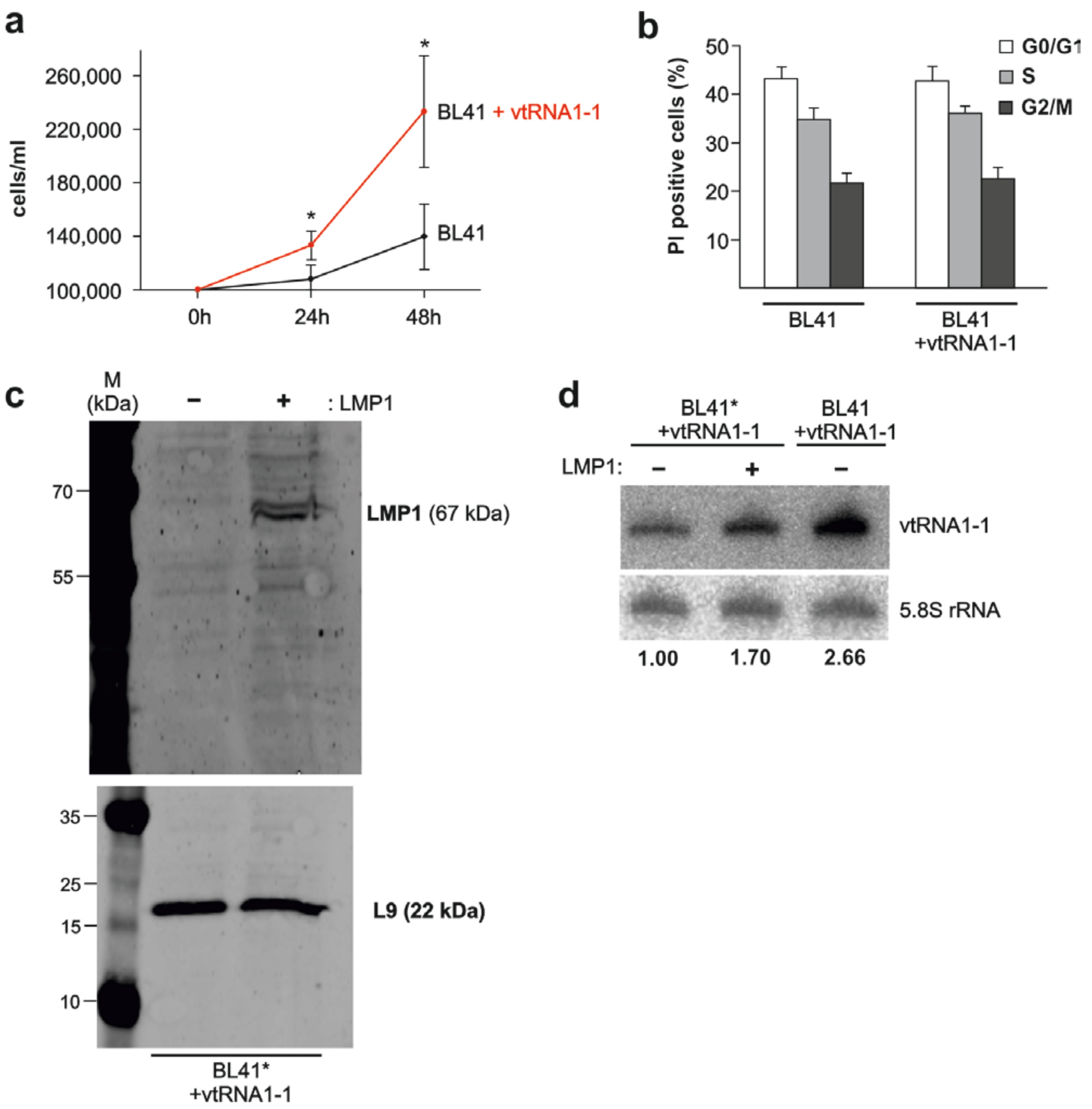


**Supplementary Figure 4:** (**a**) Growth curves showing BL41 cells (black) compared to vtRNA1-1 overexpressing BL41 cells (red). Cells were counted by a CASY cell counter (Model DT). The mean and standard deviations of three independent proliferation experiments are shown (two-tailed unpaired Student's t-test; * $P<0.05$). (**b**) The cell cycle profile of the parental BL41 cell line was compared to BL41 cells ectopically expressing vtRNA1-1. Cells were investigated by FACS analysis after a propidium iodide (PI) stain. **(c, d)** Of note, the vtRNA1-1 levels in the overexpressing BL41 cell line could not be further increased by either EBV infection or LMP1 overexpression. However, a sub-clonal cell line of BL41 ectopically expressing vtRNA1-1 to a lesser extent (BL41* + vtRNA1-1) was responsive to LMP1 expression. This cell line was transfected with the LMP1 expression construct and the levels of LMP1 (c) and vtRNA1-1 (d) were monitored by western and northern blot analyses, respectively. In (d) the levels of vtRNA1-1 were quantified and compared to the levels seen in the sub-clonal cell line BL41*+vtRNA1-1. In (d) the cell line named BL41 + vtRNA1-1 corresponds to the one otherwise used throughout this manuscript. Positions of molecular weight markers (M) are indicated on the left. The ribosomal protein L9 and the 5.8S rRNA served as internal loading controls.

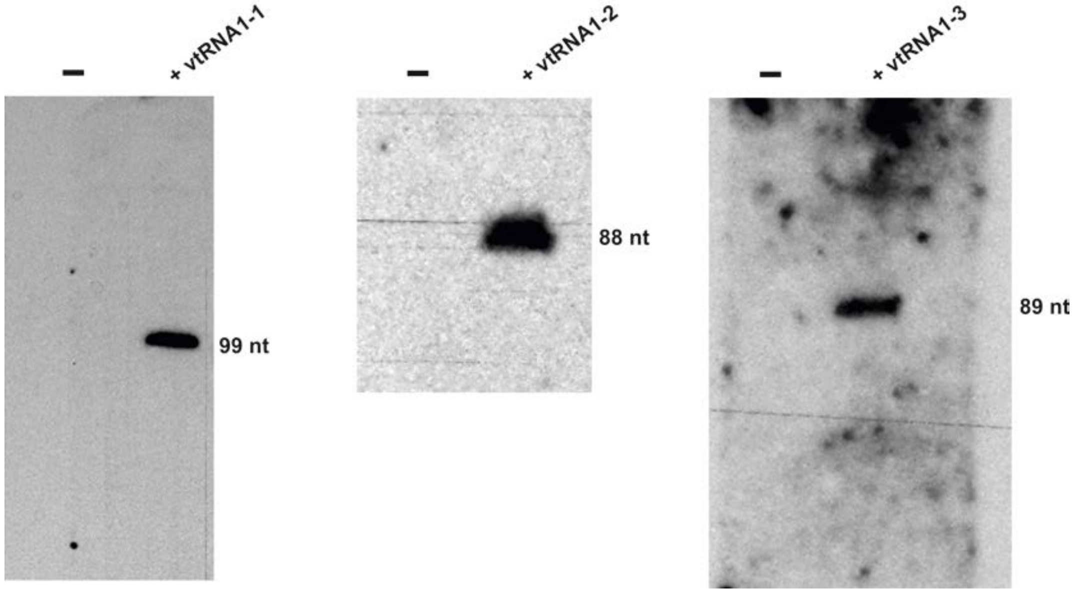


**Supplementary Figure 5: Uncropped northern blots assaying the expression levels of human vtRNAs.** Northern blot analysis reveals the amount of ectopically expressed vtRNA1-1 (99 nt long) , 1-2 (88 nt long), and 1-3 (89 nt long) in BL41 cells.

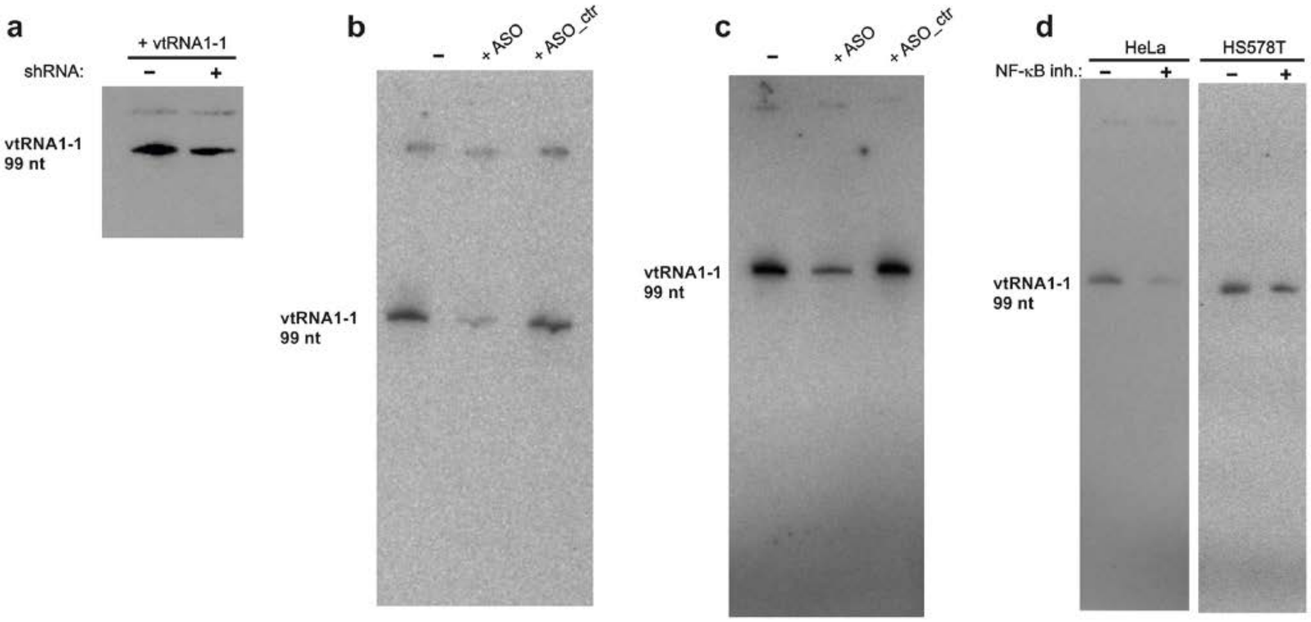


**Supplementary Figure 6: Uncropped northern blots to assess the dose dependence of vtRNA1-1 levels on the impact of apoptosis resistance. (a)** Northern blot analyses demonstrate an shRNA-mediated knock-down of vtRNA1-1 levels in BL41 cells of ~50%. **(b)** Northern blot analysis showed a ~60% reduction of endogenous vtRNA1-1 levels in HeLa cells upon transfection of an anti-sense oligonucleotide analog (ASO). Administration of a control oligonucleotide (ASO_ctr) strand had no effect. (–) depict HeLa cells that were treated as the transfected samples, but in the absence of any oligonucleotides. **(c)** Analogous to (b), vtRNA1-1 concentrations were monitored using an anti-sense oligonucleotide analog (ASO) in the human breast cancer cell line HS578T. **(d)** In the presence of the NF-κB inhibitor IKK VII, northern blot analysis showed a decreased vtRNA1-1 expression level in HeLa and HS578T cells.

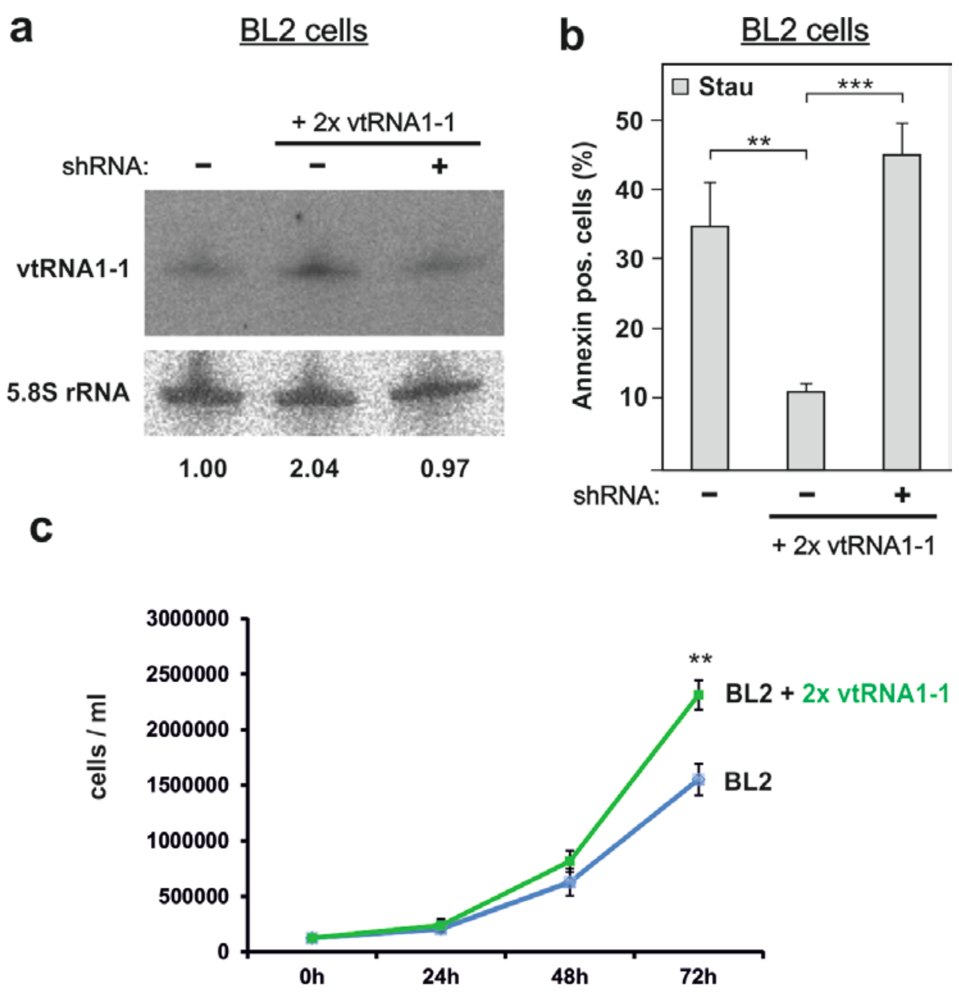


**Supplementary Figure 7: vtRNA1-1 levels affect apoptosis resistance and cell proliferation in BL2 cells.** **(a)** The endogenous vtRNA1-1 levels in BL2 cells were further increased by introducing additional vtRNA1-1 gene copies during two rounds of lentiviral transfections (+ 2x vtRNA1-1). Furthermore an shRNA-mediated knock-down of vtRNA1-1 levels in these cells was performed and the intracellular vtRNA1-1 concentrations assessed by northern blot analyses. 5.8S rRNA serves as internal loading control. The endogenous levels of vtRNA1-1 in untreated BL2 cells (-) was taken as 1.00 and compared to BL2 cells ectopically overexpressing vtRNA1-1 (+ 2x vtRNA1-1) in the absence or presence of shRNAs. **(b)** BL2 cells without (-) or containing additional vtRNA1-1 gene copies were treated with staurosporine (Stau) and the amount of apoptotic cells was determined by flow cytometry analysis after an annexin V stain. The data represent the mean and standard deviations of three independent experiments. The numbers of apoptotic cells in the untreated controls were below 20% and were subtracted from each experiment. **(c)** Growth curves showing BL2 cells (blue) compared to vtRNA1-1 overexpressing BL2 cells (green). The mean and standard deviations of three independent proliferation experiments are shown. *P* values were determined using the two-tailed unpaired Student's t-test (** *P*<0.01).

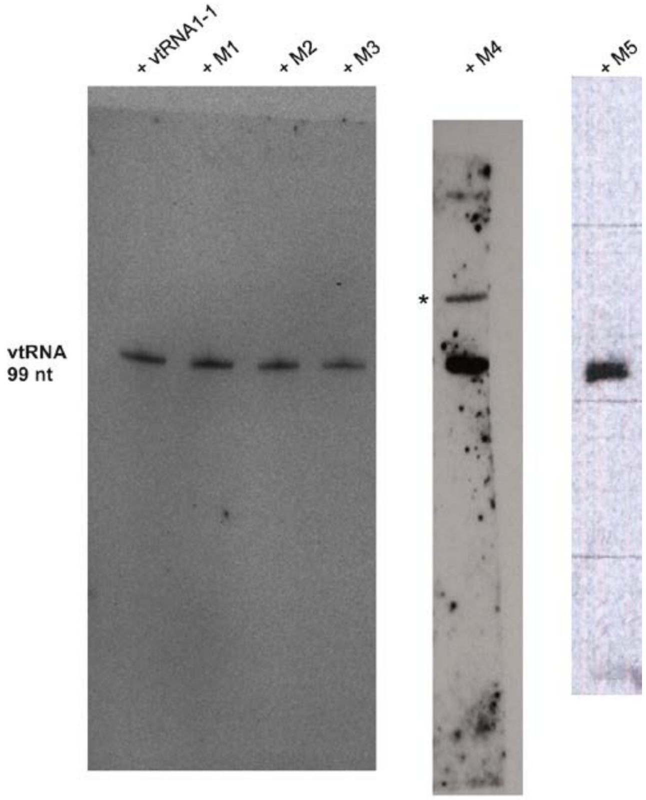


**Supplementary Figure 8: Uncropped northern blots showing expression levels of mutant versions of vtRNA.** The expression levels of mutant vtRNAs M1 to M5 in BL41 were analyzed by northern blot analyses with an oligonucleotide able to detect all vtRNA variants. The asterisk depicts a signal from a previous northern blot hybridization using the same nylon membrane but a radiolabeled probe targeting 5.8S rRNA.

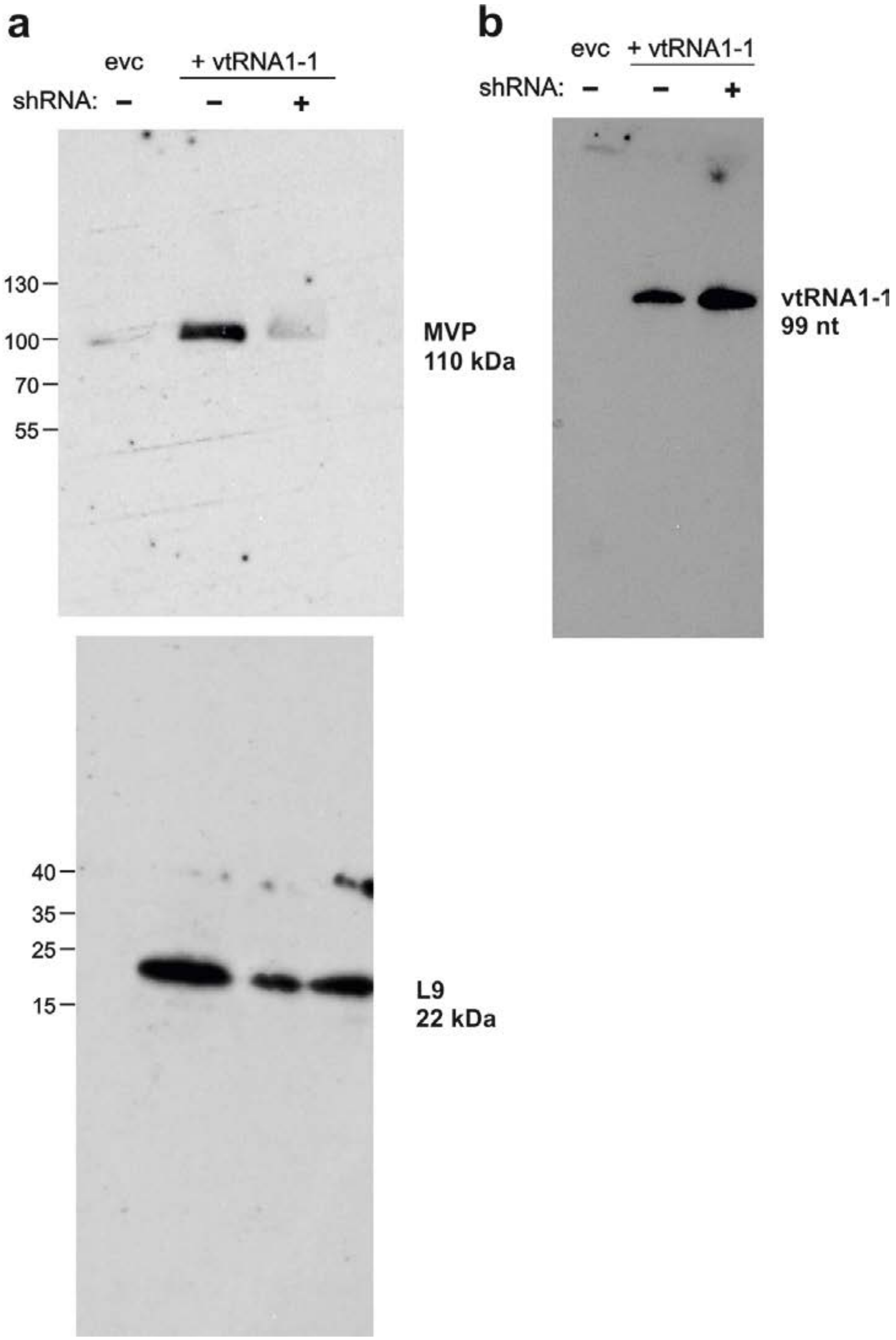


**Supplementary Figure 9: Uncropped western and northern blots monitoring levels of MVP and vtRNA1-1 upon MVP knock-down. (a)** MVP levels in BL41 cells treated with the empty vector control (evc) or ectopically expressing vtRNA1-1 in the absence (+vtRNA1-1) or presence of shRNA directed against the MVP were probed by western blot analyses. Positions of molecular weight markers (in kDa) are indicated on the left. **(b)** The expression levels of vtRNA1-1 in the cells shown in (a) were assessed by northern blotting. Ribosomal protein L9 served as loading control.

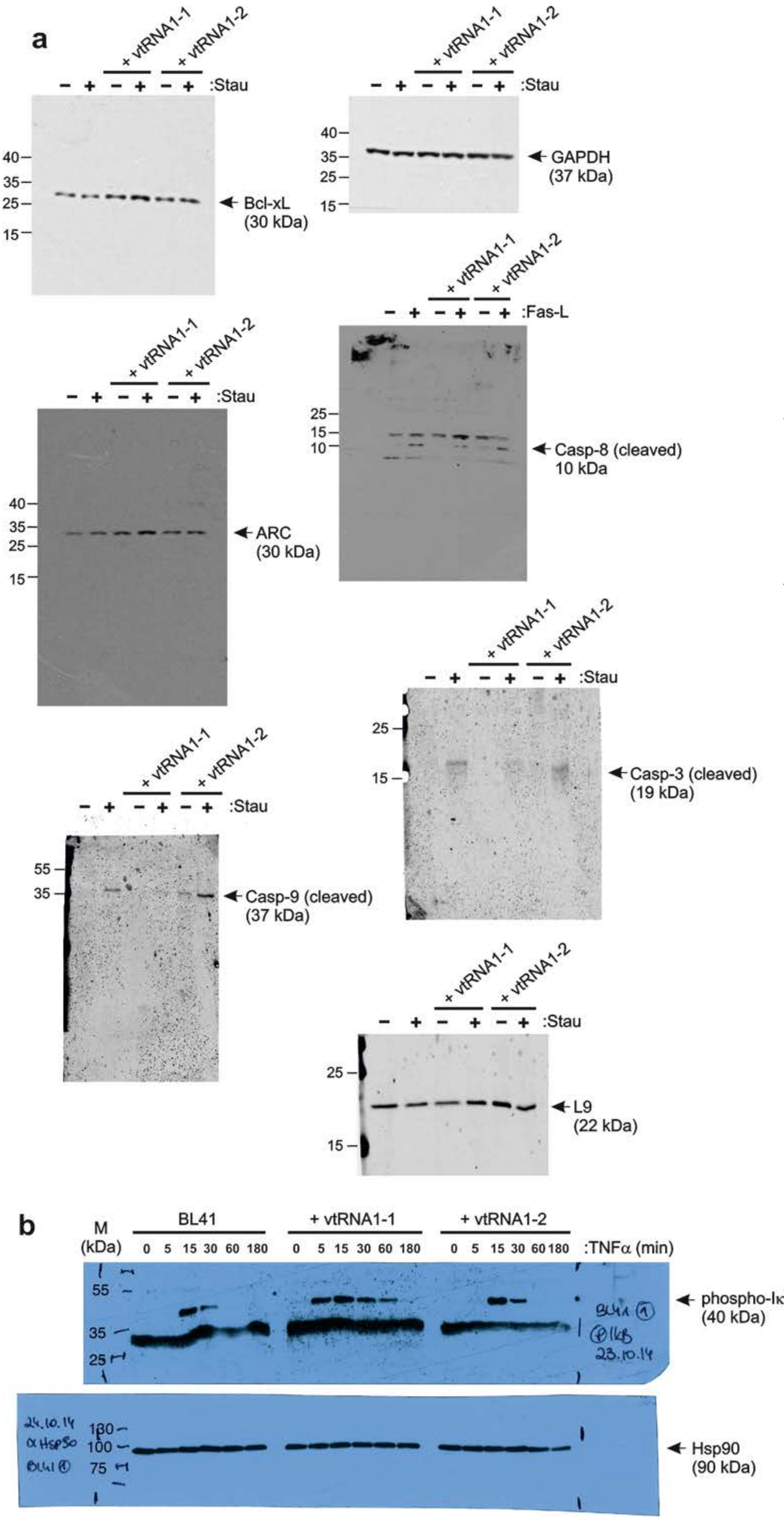


**Supplementary Figure 10: vtRNA1-1 modulates both the intrinsic and the extrinsic apoptosis pathway.** (**a**) Uncropped western blots showing the protein levels of Bcl-xL, ARC, and the cleaved caspases Casp-9, and Casp-3 in BL41 cells expressing vtRNA1-1 or vtRNA1-2 in the absence (-) or presence (+) of staurosporine (Stau) . Cleavage of Casp-8 was monitored after Fas-L treatment. Positions of molecular weight markers (in kDa) are indicated on the left. **(b)** Uncropped western blots to monitor the kinetics and levels of phosphorylated IκB expression in BL41 cells, or in BL41 cells expressing vtRNA1-1 or vtRNA1-2, respectively, as a function of TNFα incubation (in minutes). Hsp90 served as loading control. Molecular weight marker (M) is indicated on the left.

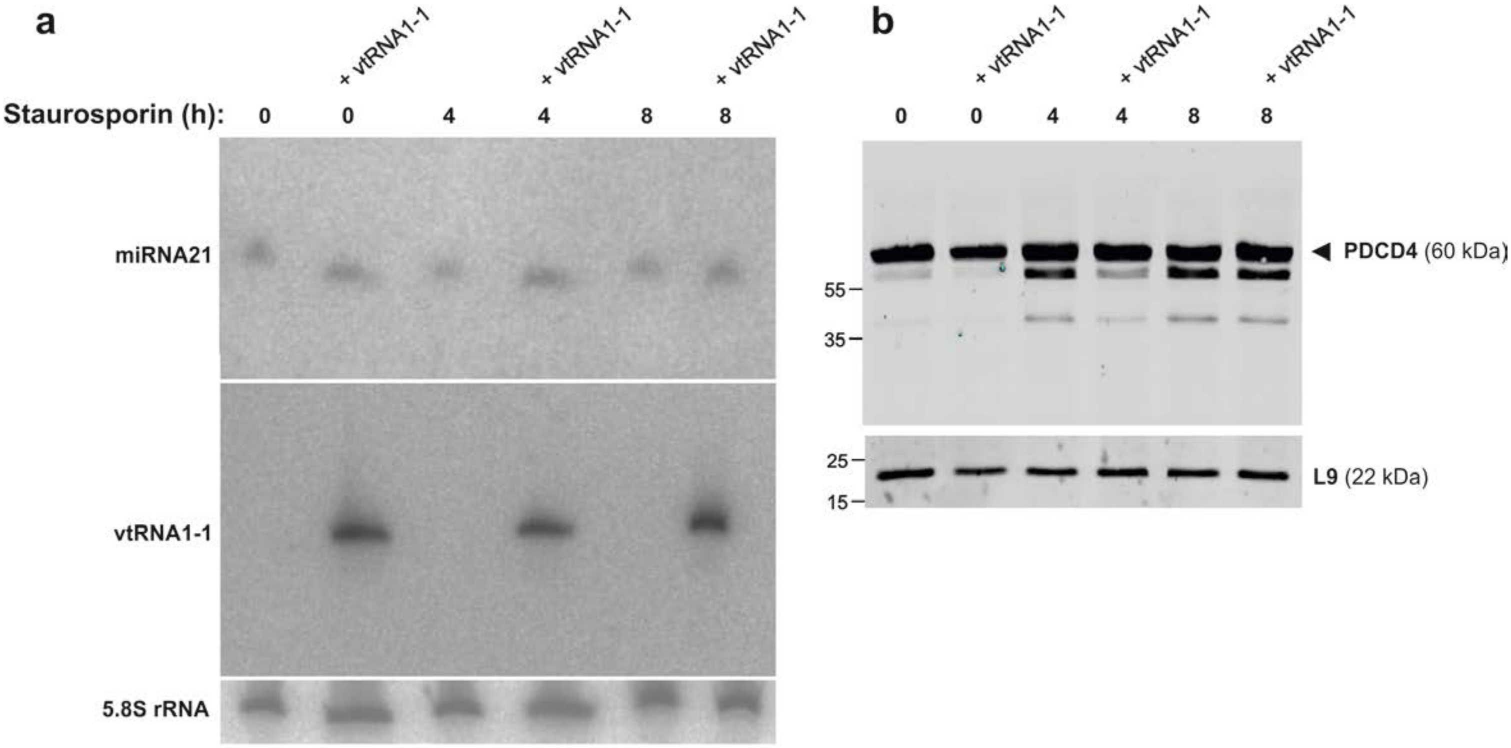


**Supplementary Figure 11: miRNA21 is not involved in vtRNA1-1 mediated apoptosis resistance.**

**(a)** vtRNA1-1 expression and/or treatment with staurosporine have no effect on miRNA21 levels in BL41 cells. 5.8S rRNA serves as loading controls for the shown northern blot. **(b)** In support of the unchanged miRNA21 levels, one of the validated cellular targets for miRNA21 (PDCD4, a protein involved in the intrinsic apoptosis pathway) was not affected in the cells under investigation. Ribosomal protein L9 served as internal loading control for the western blot. Positions of molecular weight markers (in kDa) are indicated on the left.

**Supplementary Table 1:** Quantitative PCR-array of 84 key marker genes involved in apoptosis. 'Fold changes' were calculated using the comparative $2^{-(\Delta\Delta ct)}$ method where BL41 served as calibrator and BL41 expressing vtRNA1-1 as target. The mean of two technical replicates is shown.

| | fold change (mean) | | fold change (mean) |
|---|---|---|---|
| ABL1 | 1.04 | LTBR | not detectable |
| AIFM1 | 1.28 | MCL1 | 1.31 |
| AKT1 | 1.21 | NAIP | 1.12 |
| APAF1 | 1.24 | NFKB1 | 1.49 |
| BAD | 1.03 | NOD1 | 1.20 |
| BAG1 | 1.58 | NOL3 | 3.94 |
| BAG3 | 1.53 | PYCARD | 1.16 |
| BAK1 | 1.33 | RIPK2 | 1.01 |
| BAX | 1.06 | TNF | 2.12 |
| BCL10 | 1.23 | TNFRSF10A | 1.38 |
| BCL2 | 1.07 | TNFRSF10B | 2.48 |
| BCL2A1 | 2.13 | TNFRSF11B | 4.81 |
| BCL2L1 (BCLXL) | 2.21 | TNFRSF1A | 1.03 |
| BCL2L10 | 1 | TNFRSF1B | not detectable |
| BCL2L11 | 1.39 | TNFRSF21 | 1.22 |
| BCL2L2 | 1.02 | TNFRSF25 | 1.40 |
| BFAR | 1.14 | TNFRSF9 | not detectable |
| BID | 1.50 | TNFSF10 | 3.13 |
| BIK | 1.64 | TNFSF8 | 3.71 |
| BIRC2 | 1.56 | TP53 | 1.04 |
| BIRC3 | 1.24 | TP53BP2 | 1.41 |
| BIRC5 | 1.73 | TP73 | not detectable |
| BIRC6 | 1.16 | TRADD | 1.01 |
| BNIP2 | 1.14 | TRAF2 | 1.07 |
| BNIP3 | 1.34 | TRAF3 | 1.24 |
| BNIP3L | 1.16 | XIAP | 1.05 |
| BRAF | 1.02 | | |
| CASP1 | 1.53 | | |
| CASP10 | 2.31 | | |
| CASP14 | not detectable | | |
| CASP2 | 1.13 | | |
| CASP3 | 1.18 | | |
| CASP4 | 1.38 | | |
| CASP5 | not detectable | | |
| CASP6 | 1.41 | | |
| CASP7 | 1.18 | | |
| CASP8 | 1.32 | | |
| CASP9 | 1.07 | | |
| CD27 | 2.60 | | |
| CD40 | 1.68 | | |
| CD40LG | 1.89 | | |
| CD70 | 1.23 | | |
| CFLAR | 1.27 | | |
| CIDEA | not detectable | | |
| CIDEB | 1.43 | | |
| CRADD | 1.42 | | |
| CYCS | 1.06 | | |
| DAPK1 | not detectable | | |
| DFFA | 1.01 | | |
| DIABLO | 1.15 | | |
| FADD | 1.14 | | |
| FAS | 1.10 | | |
| FASLG | not detectable | | |
| GADD45A | 1.23 | | |
| HRK | 2.18 | | |
| IGF1R | 1.12 | | |
| IL10 | not detectable | | |
| LTA | 1.27 | | |

## Supplementary Table 2: DNA oligonucleotides used in this study

**Overexpression of EBV-encoded proteins**

| | |
|---|---|
| LMP1, M_CTAR1,M_CTAR2,MCTAR1+2 Fwd: | 5'-AAAAAGCAGGCTCCATGGAATATCCTTATGACGTTC-3' |
| LMP1, M_CTAR1, M_CTAR1+2 Rev: | 5'-CAAGAAAGCTGGGTCTTAGTCATAGTAGCTTAGCTG-3' |
| LMP1_M_CTAR2 Rev: | 5'-CAAGAAAGCTGGGTCACCGGAACCAGAAGAACCC-3' |
| LMP2A Fwd: | 5'-CAAAAAAGCAGGCTCCATGGGGGGTTCTCATCATC-3' |
| LMP2A Rev: | 5'-CAAGAAAGCTGGGTCTACAGTGTTGCGATATGGGG-3' |
| LMP2b Fwd: | 5'-G**CTCGAG**GGCGACCGCCCAGCGACCCCCG-3' (bold... XhoI restriction side) |
| LMP2b Rev: | 5'-C**ACTAGT**CAATTCAACAGGCATCTACTGAG-3' (bold... SpeI restriction side) |
| EBNA1 Fwd: | 5'-CAAAAAAGCAGGCTCCATGTCTGACGAGGGGCCAGG-3' |
| EBNA1 Rev: | 5'-CAAGAAAGCTGGGTCTCCTGCCCTTCCTCACCCTC-3' |
| EBNA2 Fwd: | 5'-GGGGACAAGTTTGTACAAAAAAGCAGGCTCCATGCCTACATTCTATCTTGC-3' |
| EBNA2 Rev: | 5'-GGGGACCACTTTGTACAAGAAAGCTGGGTCTTACTGGATGGAGGGGCGAG-3' |
| EBNA-LP Fwd: | 5'-G**GAGCTC**TTCTGAGGCGGAAAGAACCAGCTG-3' (bold... SacI restriction side) |
| EBNA-LP Rev: | 5'-C**ACTAGT**CAGACATGATAAGATACATTGATG-3' (bold... SpeI restriction side) |
| AttB1: | 5'-GGGGACAAGTTTGTACAAAAAAGCAGGCT-3' |
| AttB2: | 5'-GGGGACCACTTTGTACAAGAAAGCTGGGT-3' |

underlined... attB sequence (Gateway cloning)

**Real time PCR NF-kB targets**

| | |
|---|---|
| BclXl_fwd: | 5'-GGAACAATGCAGCAGCCGAG -3' |
| BclXl_rev: | 5'-GTAGAGTGGATGGTCAGTGT-3' |
| c-FLIP_fwd: | 5'-GACAGAGCTTCTTCGAGACAC-3' |
| c-FLIP_rev: | 5'-GCTCGGGCATACAGGCAAAT-3' |
| RT_IL6_fwd: | 5'-GGCACTGGCAGAAAACAACC-3' |
| RT:IL6_rev: C | 5'-GCAAGTCTCCTCATTGAATC-3' |
| RT_TBP_fwd: | 5'-TGCACAGGAGCCAAGAGTGAA-3' |
| RT_TBP_rev: | 5'-CACATCACAGCTCCCCACCA-3' |

**ChIP**

| | |
|---|---|
| vt1-1_promoter_fwd | 5'-GGGGTTGGAATGTTTATCTAGTTTA-3' |
| vt1-1_promoter_rev | 5'-ACTTCGTGATCAAATCGACTTTTA-3' |
| vt1-2_promoter_fwd | 5'-CTCTAACAAAAAAAATTTAAAAAGC-3' |
| vt1-2_promoter_rev | 5'- GGCGTCTTTACAAGGTGTAAATTG-3' |

**EMSA**

| | |
|---|---|
| EMSA_fwd | 5'-GCGCAGAGTCTGGAAGCGAAGGAG-3' |
| EMSA_rev | 5'-GCAGTGAAAAGGACTGGAGAGCTC-3' |

**Oligonucleotides used for northern blot analysis**

| | |
|---|---|
| vtRNA 1-1: | 5'-GCTTGTTTCAATTAAAGAACTGTCG-3' |
| vtRNA 1-2: | 5'-AGGTGGTTACAATGTACTCGAAG-3' |
| vtRNA 1-3: | 5'-GAGGTGGTTTGATGACACGCGAA-3' |
| vtRNA 2-1: | 5'-TCCGGCATGAGGAGGTAACCGC-3' |
| vtRNA variants | 5'-CGCTGAGCTAAAGCCAGCC-3' |
| 5.8S rRNA | 5'-TCCTGCAATTCACATTAATTCTCGAGCTAGC-3' |

**Vault RNA overexpression**

| | |
|---|---|
| 5'hvg1-big: | 5'-GGTGAACATGCTGTAGGTACTGGTATTATCTTCTG-3' |
| 3'hvg1-big: | 5'-AGGAAGCAAGCAGTGAATAACAGTACCAGTCTAGG-3' |
| hvg 1-2: | 5'-GGAATTCGAGTAACAGGAAGTTTCACTGGGC-3' |
| hvg 1-4: | 5'-CGGATCCGCAGTGAAAAGGACTGGAGAGCTC-3' |

**vtRNA Mutants**

| | |
|---|---|
| M1 Fwd: | 5'-CGAACAACCCAGACAGGTTGCTTGTTTCAATTAAAGAACTGTCGAAG-3' |
| M1 Rev: | 5'-GCAACCTGTCTGGGTTGTTCGAGACCCGCGGGCGCTCTCCAGCTCTTTT-3' |
| M2 Fwd: | 5'-CCGCGGGTCTCGAACAACCCAGACAGGTTGCTTGTTTCAAT-3' |
| M2 Rev: | 5'-GGTTGTTCGAGACCCGCGGGTGCTTTCCAGTCCTTT-3' |
| M3 Fwd: | 5'-CCGCGGGTCTCGAACAACCCAGACAGGTTGCTTGTTTCAA-3' |
| M3 Rev: | 5'-GGGTTGTTCGAGACCCGCGGGTGCTCTCCAGTCCTTTT-3' |
| M4 Fwd: | 5'-GGTTACAATGTACTCGAAGTAACCGCTGAGCTAAAGCC-3' |
| M4 Rev: | 5'-TACTTCGAGTACATTGTAACCACCTCTCTGGGTGGTTCGAGACCCGCGGGCGCTCTCCA-3' |
| M5 Fwd: | 5'-GGTTCAATTAAAGAACTGTCGAAGTAACCGCTGAGC-3' |
| M5 Rev: | 5'-CGACAGTTCTTTAATTGAAACAAGCAACCTGTCTGGGTTGTTCGAGACCCGCG-3' |

**MVP knockdown**

| | |
|---|---|
| MVP_sense | 5'-**GATCT**CCGGCCCATACCACTATATCCATGTctcgagACATGGATATAGTGGTATGGGTTTTTTG**A**-3' |
| MVP_antisense | 5'-**AGCTT**CAAAAAACCCATACCACTATATCCATGTctcgagACATGGATATAGTGGTATGGGCCGG**A**-3' |

**vtRNA1-1 knockdown**

| | |
|---|---|
| vt1-1 sense | 5'-**GATC**TCCGGGGCTGGCTTTAGCTCAGCGttcaagagaCGCTGAGCTAAAGCCAGCCTTTTTTG**A**-3' |
| vt1-1 antisense | 5'-**AGCT**TCAAAAAAGGCTGGCTTTAGCTCAGCGtctcttgaaCGCTGAGCTAAAGCCAGCCCCGG**A**-3' |
| ASOvt1-1 | 5'-mU*mG*mU*mU*mU*C*A*A*T*T*A*A*A*G*A*mA*mC*mU*mG*mU-3' |
| ASOcontrol | 5'-mA*mG*mA*mG*mG*T*G*G*T*T*A*C*A*A*T*mG*mU*mA*mC*mU-3' |

underlined... termination signal
small letters...hairpin
bold...Bgl II, Hind III restriction sides respectively
m...2'-O-methyl modified ribonucleotides
*...phosphorothioate backbone

# Supplementary References


1 Kamranvar, S. A., Gruhne, B., Szeles, A. & Masucci, M. G. Epstein-Barr virus promotes genomic instability in Burkitt's lymphoma. *Oncogene* **26**, 5115-5123, doi:10.1038/sj.onc.1210324 (2007).
